# Dual role of autophagy on docetaxel-sensitivity in prostate cancer cells

**DOI:** 10.1038/s41419-018-0866-5

**Published:** 2018-08-30

**Authors:** Riccardo Cristofani, Marina Montagnani Marelli, Maria Elena Cicardi, Fabrizio Fontana, Monica Marzagalli, Patrizia Limonta, Angelo Poletti, Roberta Manuela Moretti

**Affiliations:** Department of Excellence: Dipartimento di Scienze Farmacologiche e Biomolecolari (DiSFeB), Università degli Studi di Milano, Milano, Italy

## Abstract

Prostate cancer (PC) is one of the leading causes of death in males. Available treatments often lead to the appearance of chemoresistant foci and metastases, with mechanisms still partially unknown. Within tumour mass, autophagy may promote cell survival by enhancing cancer cells tolerability to different cell stresses, like hypoxia, starvation or those triggered by chemotherapic agents. Because of its connection with the apoptotic pathways, autophagy has been differentially implicated, either as prodeath or prosurvival factor, in the appearance of more aggressive tumours. Here, in three PC cells (LNCaP, PC3, and DU145), we tested how different autophagy inducers modulate docetaxel-induced apoptosis. We selected the mTOR-independent disaccharide trehalose and the mTOR-dependent macrolide lactone rapamycin autophagy inducers. In castration-resistant PC (CRPC) PC3 cells, trehalose specifically prevented intrinsic apoptosis in docetaxel-treated cells. Trehalose reduced the release of cytochrome *c* triggered by docetaxel and the formation of aberrant mitochondria, possibly by enhancing the turnover of damaged mitochondria via autophagy (mitophagy). In fact, trehalose increased *LC3* and *p62* expression, LC3-II and p62 (p62 bodies) accumulation and the induction of LC3 puncta. In docetaxel-treated cells, trehalose, but not rapamycin, determined a perinuclear mitochondrial aggregation (mito-aggresomes), and mitochondria specifically colocalized with LC3 and p62-positive autophagosomes. In PC3 cells, rapamycin retained its ability to activate autophagy without evidences of mitophagy even in presence of docetaxel. Interestingly, these results were replicated in LNCaP cells, whereas trehalose and rapamycin did not modify the response to docetaxel in the *ATG5-*deficient (autophagy resistant) DU145 cells. Therefore, autophagy is involved to alter the response to chemotherapy in combination therapies and the response may be influenced by the different autophagic pathways utilized and by the type of cancer cells.

## Introduction

In men, prostate cancer (PC) is the second most common form of cancer and one of the leading causes of cancer death. PC is initially hormone-dependent and androgen deprivation therapy (ADT) is the preferred treatment also used for relapsed and metastatic PC patients^1^. During ADT many patients develop metastatic castration-resistant PC (CRPC)^2–4^, and they are treated with chemotherapeutic agent, like docetaxel^5–7^ which, despite clinical benefits, may induce docetaxel-resistance^8^, possibly due to an aberrant autophagy response^9^.

Macroautophagy (hereafter autophagy) is a conserved degradative pathway in which proteins or cytoplasmic components are engulfed into autophagosomes that fuse with lysosomes, for their degradation^10^. Generally, autophagy promotes cell survival in response to starvation or other cell stresses. Autophagy has been implicated in the aetiology of cancer, acting as either prodeath or prosurvival factor depending on type and stage of cancer considered. Oncosuppressive autophagy functions relate to decreased accumulation of genetic and genomic defects typical of malignant transformation. Indeed, an inefficient autophagy allows tumorigenesis and characterizes the early stage of cancer. Conversely, increased autophagy characterizes existing advanced malignancies correlating with an invasive/metastatic phenotype. Therefore, autophagy defects might facilitate cancer transformation of healthy cells but enhance autophagic responses to support cancer cells survival, proliferation and growth in adverse microenvironmental conditions^11,12^. Also during cancer treatment autophagy has a paradoxical effect related to contest, type, and stage of tumours. Autophagy may enhance cancer therapy efficacy through cell death promotion by itself (cell death type II) or in cooperation with apoptosis (cell death type I)^13–15^, and thus it is crucial to understand how autophagy regulates or is regulated by apoptosis.

Acting as a defensive stress mechanism, autophagy is also involved in chemoresistance^9,16–18^, by enhancing cell stress tolerance. Moreover, autophagy by removing damaged mitochondria (mitophagy) prevents chemotherapy-induced apoptosis^19,20^, since mitophagy increases apoptotic resistance^21,22^. Thus, the response to chemotherapy (docetaxel) may be modulated by autophagy inducers in CRPC cell lines.

The natural disaccharide trehalose is a potent autophagy inducer^23^ used to improve the clearance of misfolded proteins causing proteotoxic cell stresses in cell and animal models of neurodegenerative diseases^24–30^. However, it is unknown whether trehalose can activate autophagy in CRPC cells. The macrolide lactone rapamycin is another autophagy activator. While trehalose does not involve the mammalian target of rapamycin (mTOR)^31,32^, rapamycin specifically inhibits the mTOR pathway. The mTOR signalling represses autophagy and regulates cell growth, proliferation, survival, angiogenesis^33–36^ and is upregulated in almost 50% of PC^37^. In PC cells, rapamycin exerts cytotoxic effects^38^ and enhances radio- and chemio- sensitivity^39–41^.

In this study, we investigated the effects of trehalose and rapamycin on the docetaxel response in classical PC cell lines (LNCaP, PC3 and DU145) demonstrating that these two autophagy activators exert very different roles on docetaxel-sensitivity. Trehalose prevents intrinsic apoptosis induced by docetaxel in PC cells promoting cytoprotective mitophagy. Conversely, rapamycin induces a type II cell death, that does not alter the ability of docetaxel to trigger apoptotic response, but rather enhances the cytotoxic effect of chemotherapy. Thus, depending on the type of autophagy activation, docetaxel-induced toxicity may be differentially modulated in CRPC cells. These observations are crucial to design combination therapies to prevent cancer resistance and enhance the effects of anticancer therapies.

## Results

Here, we analysed how autophagy modulates docetaxel-sensitivity of CRPC cells, using two different known autophagy-inducing agents and we evaluated the PC3 cells autophagy response.

### Trehalose induces autophagy in PC3 cells

**Fig. 1:**
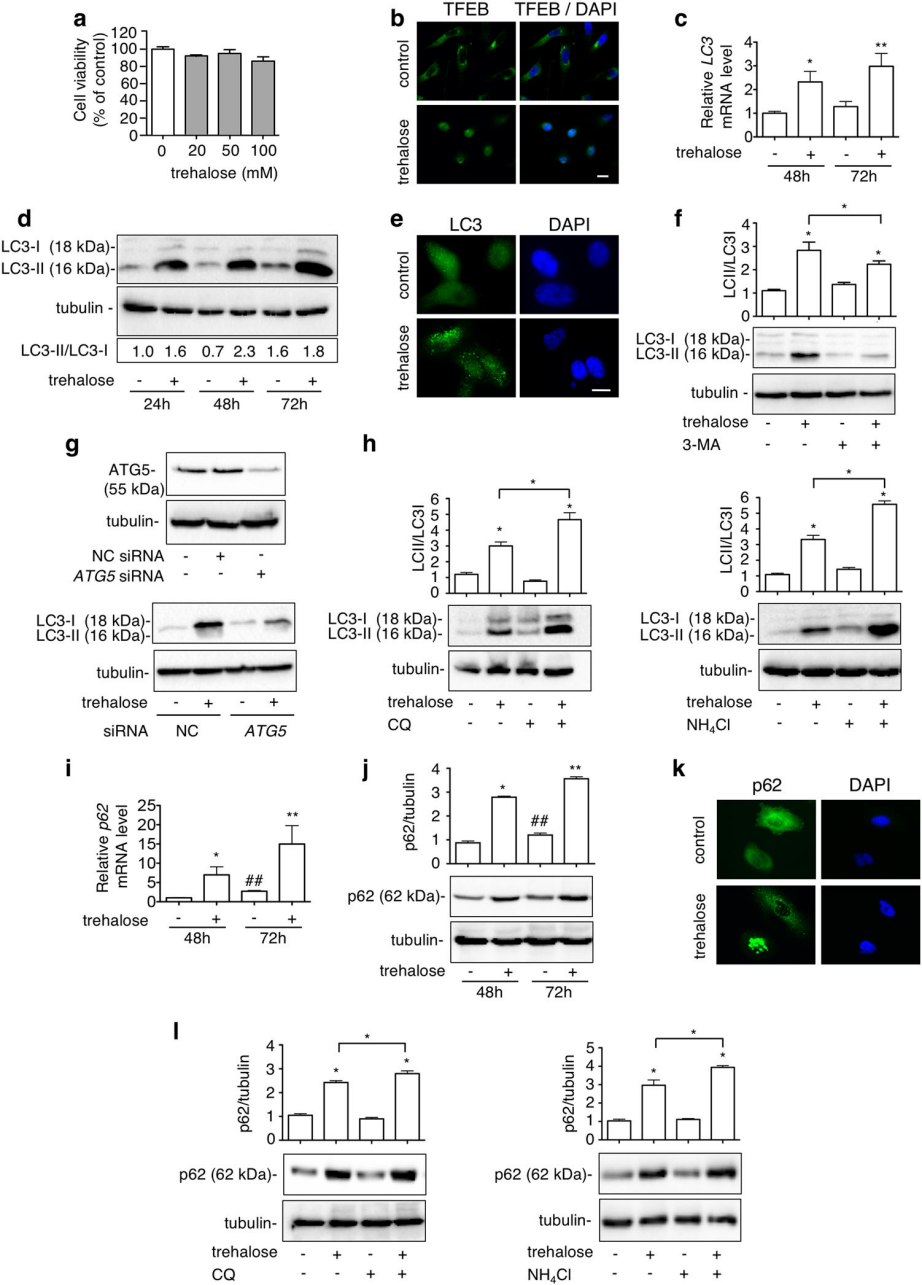
Trehalose induces autophagy in PC3 cells. **a** MTT viability assay was performed on PC3 cells treated for 48 h with 20, 50 or 100 mM trehalose. Six independent biological samples for each condition were analysed (*n* = 6), bar graph represents the mean relative cell viability ± SD. Statistical analysis was performed using Student’s *t* test. **b** TFEB localization was carried out by IF after treatment with 100 mM trehalose for 24 h. Nuclei were stained with DAPI. Scale bar, 20 μm. **c** *LC3* mRNA expression was analysed by RT-qPCR after treatment with 100 mM trehalose for 48 or 72 h. Data were normalized to the amount of *RplP0* mRNA. Bar graph represents the mean of four independent biological samples (*n* = 4) ± SD (*p < 0.01 vs. control 48 h, **p < 0.01 vs. control 72 h; Student’s *t* test). **d** Autophagy was analysed by quantification of LC3-II/LC3-I ratio by WB analysis of PC3 cells treated for 24, 48 or 72 h with 100 mM trehalose. **e** LC3 puncta was carried out by IF after treatment with 100 mM trehalose for 48 h. Nuclei were stained with DAPI. Scale bar, 20 μm. **f** PC3 cells pretreated with or without 1 mM 3-MA for 1 h were exposed to 100 mM trehalose for additional 48 h. WB analysis of LC3 was carried out. Relative optical density of LC3II/I was quantified by ImageJ software. Bar graph represents mean ± SD calculated from three independent experiments. Statistical analysis was performed by one-way ANOVA followed by Bonferroni post-test (*p < 0.05). **g** PC3 cells were transfected with 50 nM negative control (NC) or *ATG5* siRNA and WB of ATG5 was performed. Quantification of LC3 in PC3 cells transfected and treated 48 h with 100 mM trehalose was carried out. **h** PC3 cells were treated with 100 mM trehalose for 48 h and with 10 μM CQ or 2.5 mM NH_4_Cl for the last 24 h before their collection. WB shows LC3 protein levels. Relative optical density of LC3II/I was determined by ImageJ software. Bar graph represents mean ± SD calculated from three independent experiments. Statistical analysis was performed by one-way ANOVA followed by Bonferroni post-test (*p < 0.05). **i** *p62* mRNA expression levels were detected by RT-qPCR after treatment with 100 mM trehalose for 48 or 72 h. Data were normalized to the amount of *RplP0* mRNA. Bar graph represents the mean of four biological independent samples (*n* = 4) ± SD (*p < 0.05 vs. control 48 h, **p < 0.01 vs. control 72 h; #*p* < 0.05 vs. control 48 h, Student’s *t* test). **j** PC3 cells were treated with 100 mM trehalose for 48 or 72 h. Twenty micrograms of protein extract was analysed by WB against p62. The quantification results were calculated over three individual experiments. Statistical analysis was performed by one-way ANOVA with Bonferroni post-test (*p < 0.05 vs. control 48 h, **p < 0.05 vs. control 72 h; #*p* < 0.05 vs. control 48 h). **k** p62 localization was analysed by IF with anti-p62 antibody followed by FITC-conjugated secondary antibody in cells treated with 100 mM trehalose for 48 h. Nuclei were stained with DAPI. Scale bar, 20 μm. **l** PC3 cells were treated with 100 mM trehalose for 48 h and with 10 μM CQ or 2.5 mM NH_4_Cl for the last 24 h before their collection. p62 levels were analysed by WB. Bar graph represents quantification mean ± SD calculated from three independent experiments. Statistical analysis was performed by one-way ANOVA with Bonferroni post-test (*p < 0.05)

Trehalose effect on cell viability was tested in PC3 cells (20–100 mM) without observing modifications (Fig. 1a). Based on the literature, 100 mM of trehalose was selected to activate autophagy^31,42^ which was analysed evaluating nuclear translocation of transcription factor EB (TFEB) (one of the master regulators of autophagy that induces the expression of several autophagy and lysosome-related genes)^43^, mRNA expression and protein levels and distribution of two classical autophagy markers: microtubule-associated protein1A/1B-light chain 3 (LC3) and the sequestosome-1 (SQSTM1) or p62^44^. At this concentration, in PC3 cells trehalose induced nuclear translocation of TFEB (Fig. 1b) and enhanced LC3 mRNA expression after 48 and 72 h (Fig. 1c), suggesting that autophagy was activated. During autophagy activation, LC3 is converted from its diffuse cytoplasmic LC3-I form to LC3-II, the lipidated form recruited to the nascent autophagosomes membrane. Therefore, we analysed the LC3-II/LC3-I ratio in western blot (WB) and its intracellular distribution (from diffuse to punctate) by immunofluorescence microscopy (IF). We found that trehalose increased LC3-II levels (Fig. 1d), and induced LC3 puncta formation (Fig. 1e). Since autophagosome-anchored LC3-II is cleared from cells by autophagy, we quantified trehalose-mediated activation of autophagy after autophagy flux inhibition with 3-methyladenine (3-MA) added 1 h before trehalose treatment. As shown in Fig. 1f, the levels of trehalose-induced LC3-II were greatly reduced by 3-MA. The effect of trehalose on autophagy was confirmed to be specific by silencing the *ATG5* gene^45^, whose protein product is required to activate autophagy (Fig. 1g). Then, we inhibited the clearance of autophagosomes and amphisomes with chloroquine (CQ) and NH_4_Cl (two lysosomal lumen alkalizers which neutralize the acidic pH inactivating lysosomal degrading enzymes)^46^. As shown in Fig. 1h, both CQ and NH_4_Cl increased LC3-II levels induced by trehalose suggesting that in PC3 cells trehalose induces and sustains a normal autophagy flux. Autophagic flux activation was also analysed by the overexpression of GFP-LC3 reporter vector with or without autophagosome-lysosome fusion inhibition (Fig. S1a)^47^.

Next, we analysed the autophagy adaptor p62, which binds polyubiquitinated proteins engulfing them into autophagosomes^44^. As reported for LC3, p62 expression is upregulated during autophagy activation and it is degraded by autophago-lysosomes, but when the autophagic flux is insufficient or blocked p62 accumulates into liquid droplets called “p62 bodies”. p62 is upregulated in different cancer types, and in CRPC correlates with tumour progression and resistance to therapies^48,49^. In PC3 cells we found that trehalose increases p62 mRNA and protein levels both at 48 and 72 h after treatment (Fig. 1i, j). IF analysis showed that trehalose-treated cells contain several “p62 bodies” while p62 has a diffuse cytosolic localization in control cells (Fig. 1k). Trehalose cotreatment with CQ or NH_4_Cl increased p62 levels compared to trehalose-treated cells (Fig. 1l). Moreover, trehalose treatment increased the clearance of an elongated polyQ containing protein (Fig. S1b) indicating that autophagic flux is active. In fact, it normally accumulates as insoluble species in the cells as consequence of autophagy impairment^28,30^. Thus, trehalose induces autophagy in PC3 cells  and LC3 and p62 protein levels heightened because their synthesis is greater than their degradation.

### Rapamycin induces autophagy in PC3 cells

**Fig. 2:**
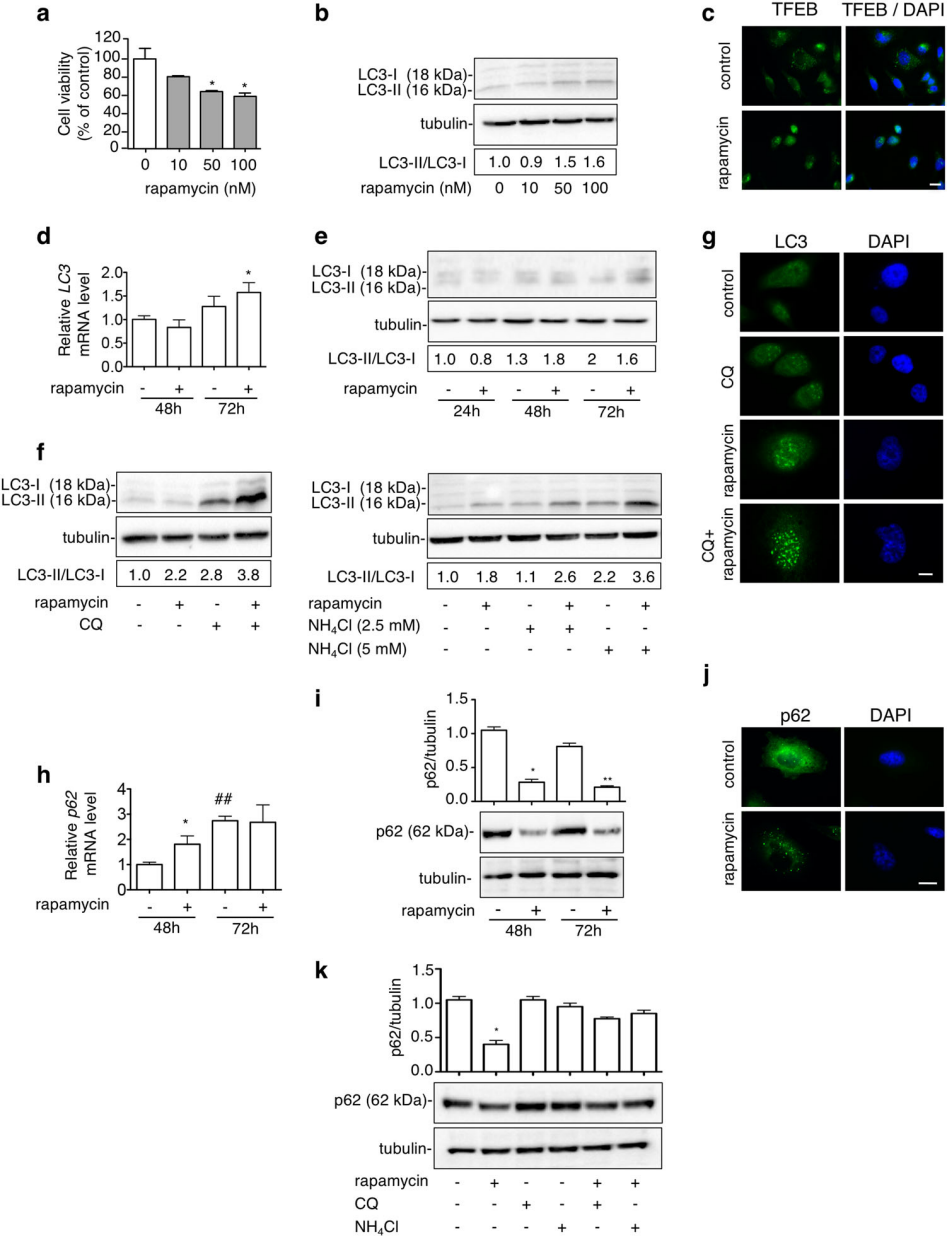
Rapamycin induces autophagy in PC3 cells. **a** PC3 cells were treated for 48 h with 10, 50 or 100 nM rapamycin. MTT viability assay was performed. Data are mean ± SD of six independent biological samples (*n* = 6). Statistical analysis was performed using Student’s *t* test (*p < 0.05 vs. control). Each experiment was repeated three times. **b** WB analysis of LC3 was performed with lysate of cells treated for 48 h with different doses of rapamycin (10, 50, 100 nM). Fifteen micrograms of protein extract was loaded in SDS-gel electrophoresis. Detection of autophagy was analysed by quantification of LC3-II/LC3-I ratio. Relative optical density was determined by ImageJ software. Experiments were performed independently three times and a representative blot is shown. **c** TFEB localization was carried out by IF after treatment with 100 nM rapamycin for 48 h. Nuclei were stained with DAPI. Scale bar, 20 μm. **d** *LC3* mRNA expression levels were analysed by RT-qPCR after treatment with 100 nM rapamycin for 48 or 72 h. Data were normalized to the amount of *RplP0* mRNA. Data are mean ± SD of four independent biological samples (*n* = 4). Statistical analysis was performed using Student’s *t* test (*p < 0.05 vs. control 72 h). **e** WB analysis of LC3 was performed with lysate of cells treated for 24, 48 or 72 h with 100 nM rapamycin. Detection of autophagy was analysed by quantification of LC3-II/LC3-I ratio. Relative optical density was determined by ImageJ software. Experiments were performed independently three times and a representative blot is shown. **f** Cells were treated with 100 nM rapamycin for 48 h and CQ (10 μM) or NH_4_Cl (2.5 mM or 5 mM) for the last 24 h before their collection. LC3 levels were analysed by WB and relative optical density of LC3II/I was determined by ImageJ software. Experiments were performed independently three times and a representative blot is shown. **g** LC3 puncta were analysed by IF utilizing anti-LC3 antibody followed by FITC-conjugated secondary antibody. Cells were treated with 100 nM rapamycin alone for 48 h or in combination with 10 μM CQ for the last 24 h. Nuclei were stained with DAPI. Scale bar, 20 μm. **h** *p62* mRNA expression levels were detected by RT-qPCR after treatment with 100 nM rapamycin for 48 or 72 h. Data were normalized to the amount of *RplP0* mRNA. Data are mean ± SD of four independent biological samples (*n* = 4). Statistical analysis was performed using Student’s *t* test (*p < 0.01 vs. control 48 h, ##*p* < 0.01 vs. control 48 h). **i** Cells were treated with 100 nM rapamycin for 48 or 72 h. Twenty micrograms of protein extract was analysed by WB. The quantification results were calculated from three independent experiments. Statistical analysis was performed using one-way ANOVA followed by Bonferroni post-test (*p < 0.05 vs. control 48 h, **p < 0.05 vs. control 72 h). **j** p62 was analysed by IF using anti-p62 antibody followed by FITC-conjugated secondary antibody in cells treated with 100 nM rapamycin for 48 h. Nuclei were stained with DAPI. Scale bar, 20 μm. **k** Cells were treated with 100 nM rapamycin for 48 h and 10 μM CQ or 2.5 mM NH_4_Cl for the last 24 h before their collection. p62 levels were analysed by WB. Bands relative optical density was determined by ImageJ software. The quantification results were calculated from three independent experiments. Statistical analysis was performed by one-way ANOVA with Bonferroni post-test (*p < 0.05 vs. control)

Next, we compared the mTOR-independent trehalose-induced autophagy to that triggered via the mTOR inhibitor rapamycin. Rapamycin becomes cytotoxic at dose higher than 50 nM (Fig. 2a). We analysed rapamycin-induced autophagy activation (doses from 10 to 100 nM) and selected the best concentration of 100 nM (Fig. 2b), which is usually used in PC studies to trigger autophagy^50–52^. At this dose, in PC3 cells rapamycin induced nuclear translocation of TFEB (Fig. 2c) and increased LC3 mRNA only after 72 h (Fig. 2d) and did not affect the LC3-II levels and the LC3-II/LC3-I ratio (Fig. 2e). Conversely, both parameters were increased after cotreatment with CQ and NH_4_Cl indicating that rapamycin induces the autophagic flux in PC3 cells (Fig. 2f). LC3 distribution confirmed a punctate staining in rapamycin-treated cells (with or without CQ) indicative of LC3 accumulation into autophagosomes (Fig. 2g), suggesting an elevated LC3 turnover upon rapamycin treatment in basal condition. In parallel, rapamycin induced p62 mRNA expression only at 48 h (Fig. 2h), while p62 protein levels and distribution showed a reduction of p62 protein and the appearance of p62 bodies (Fig. 2i, j). CQ or NH_4_Cl treatments counteracted rapamycin-induced p62 reduction (Fig. 2k). Therefore, rapamycin activates autophagy and autophagic flux rapidly degrades LC3 and p62 via autophago-lysosomes.

**Fig. 3:**
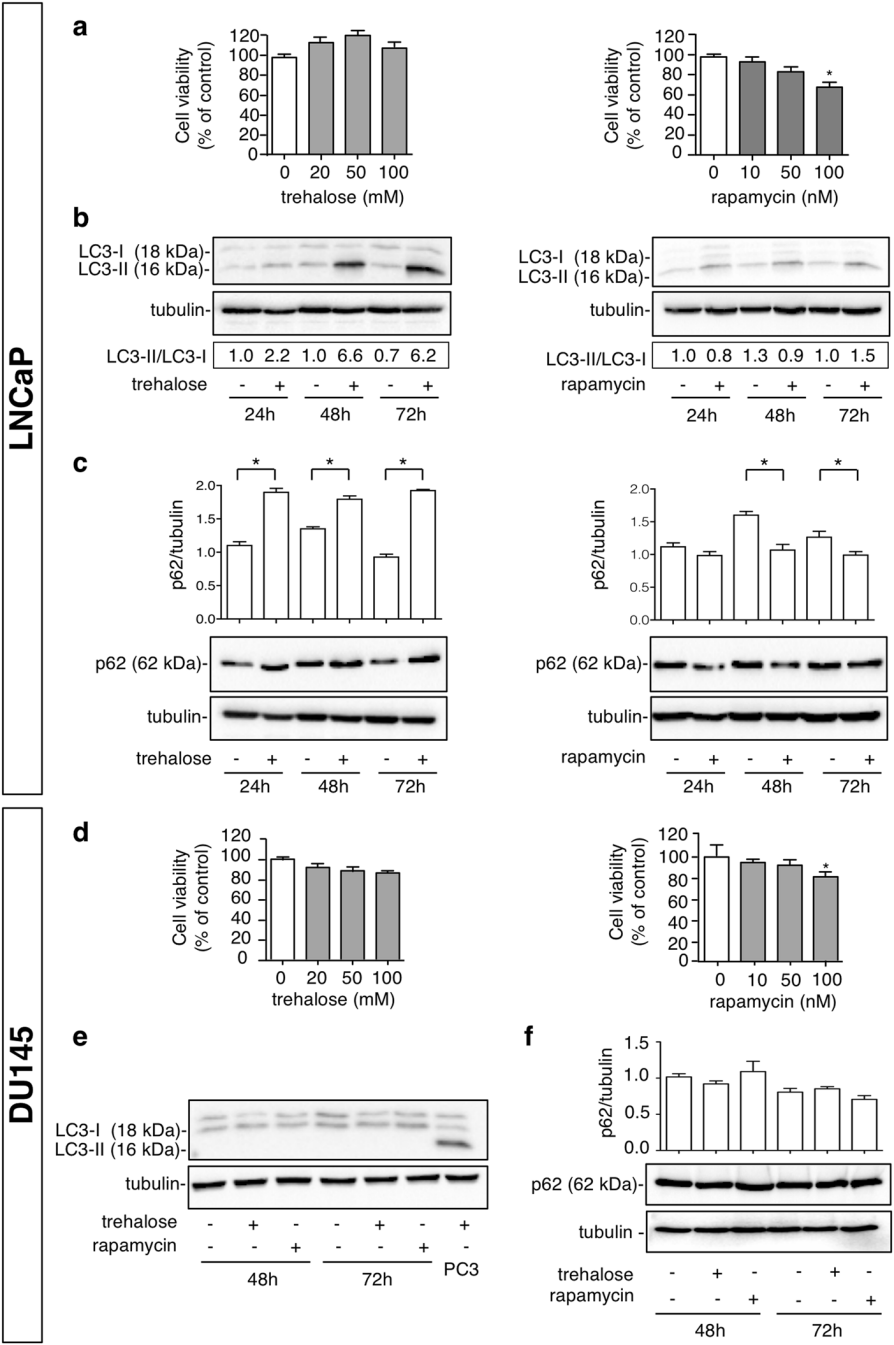
Trehalose and rapamycin induce autophagy in LNCaP cells, but do not induce autophagy in DU145 cells. **a** LNCaP cells were treated with 20, 50,100 mM trehalose or with 10, 50, 100 nM rapamycin for 48 h. MTT assays were performed. Data are mean ± SD of six independent biological samples (*n* = 6). Each experiment was repeated three times. Statistical analysis of data was performed by Dunnet test (*p < 0.05 vs. control). **b** LNCaP cells were treated for 24, 48 or 72 h with 100 mM trehalose or 100 nM rapamycin and WB analysis of LC3 was performed. Detection of autophagy was analysed by quantification of LC3-II/LC3-I ratio. Relative optical density was determined by ImageJ software. Experiments were performed independently three times and a representative blot is shown. **c** LNCaP cells were treated for 24, 48 or 72 h with 100 mM trehalose or 100 nM rapamycin and WB analysis of p62 was performed. Tubulin was used as loading control. Bar graph represents mean optical density ± SD of p62 levels. The experiments were performed independently three times. Statistical analysis was performed by one-way ANOVA with Bonferroni post-test (*p < 0.05). **d** DU145 cells were treated with 20, 50,100 mM trehalose or with 10, 50, 100 nM rapamycin for 48 h. MTT assays were performed. Data are mean ± SD of six independent biological samples (*n* = 6). Each experiment was repeated three times. Statistical analysis of data was performed by Dunnet test (*p < 0.05 vs. control). **e** DU145 cells were treated for 48 or 72 h with 100 mM trehalose or 100 nM rapamycin. WB analysis of LC3 was performed. Protein extract of PC3 cells treated with 100 mM trehalose for 48 h was utilized as a positive control, tubulin was used as loading control. **f** DU145 cells were treated for 48 or 72 h with 100 mM trehalose or 100 nM rapamycin. WB analysis of p62 was performed. Tubulin was used as loading control. Bar graph represents mean optical density ± SD of p62 levels. The experiments were performed independently three times. Statistical analysis was performed by one-way ANOVA with Bonferroni post-test.

We also evaluated the effect of trehalose and rapamycin in another classical model of PC cells, LNCaP cells, and we found a complete overlap between the two cell models on trehalose and rapamycin cell viability (Fig. 3a) and autophagy induction (Fig. 3b, c). Next, we tested whether these effects require a functional autophagic activation using DU145 cells lacking expression of functional ATG5^45^. In DU145 cells trehalose and rapamycin exert the same effect as in the PC3 and LNCaP cells on cell viability (Fig. 3d), but as expected autophagy is not activated by trehalose and rapamycin (Fig. 3e, f).

### Docetaxel induces apoptosis, autophagy, and mitochondrial fission in PC3 cells

**Fig. 4:**
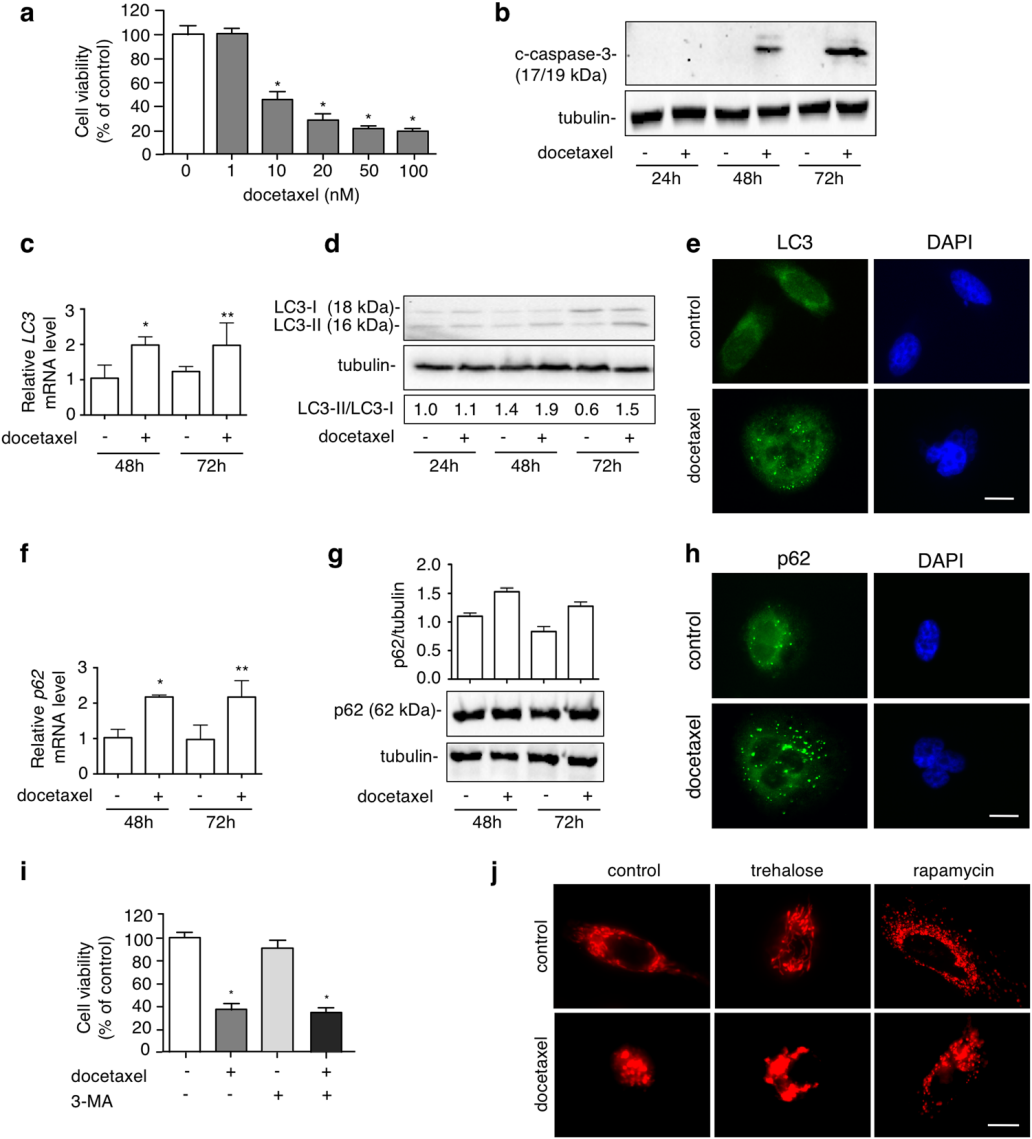
Docetaxel induces apoptosis, autophagy and mitochondrial fission in PC3 cells. **a** MTT viability assay was performed on PC3 cells treated with 1, 10, 20, 50, 100 nM docetaxel for 48 h. Six independent biological samples for each condition were analysed (*n* = 6), bar graph represents the mean relative cell viability ± SD. Statistical analysis was performed using Dunnet test (*p < 0.05 vs. control). **b** PC3 cells were treated with 20 nM docetaxel for 24, 48 or 72 h. WB shows the levels of cleaved-caspase-3 (c-caspase-3), 35 μg of protein extract was loaded. Experiments were performed independently three times and a representative blot is shown. **c** *LC3* mRNA expression levels were analysed by RT-qPCR after treatment with 20 nM docetaxel for 48 or 72 h. Data were normalized to the amount of *RplP0* mRNA. Data are mean ± SD of four independent biological samples (*n* = 4). Statistical analysis was performed using Student’s *t t*est (*p < 0.05 vs. control 48 h, **p < 0.01 vs. control 72 h). **d** To analyse the autophagy activation, cells were treated with 20 nM docetaxel for 24, 48 or 72 h. LC3 expression was evaluated by WB. Quantification of LC3-II/LC3-I ratio was performed by the measure of bands optical density by ImageJ software. The experiments were performed independently three times and a representative blot is shown. **e** IF shows LC3 distribution in PC3 cells after 48 h of 20 nM docetaxel treatment. Nuclei were stained with DAPI. Scale bar, 20 μm. **f** *p62* mRNA expression levels were detected by RT-qPCR after treatment with 20 nM docetaxel for 48 or 72 h. Data were normalized to the amount of *RplP0* mRNA. Data are mean ± SD of four independent biological samples (*n* = 4). Statistical analysis was performed using Student’s *t* test (*p < 0.01 vs. control 48 h, **p < 0.01 vs. control 72 h). **g** WB shows p62 protein expression levels after treatment with docetaxel 20 nM for 48 or 72 h. Three independent experiments were analysed; bar graph represents p62/tubulin mean optic density ± SD. Statistical analysis was performed by Student’s *t* test. **h** p62 IF analysis was done after 48 h of docetaxel treatment. Nuclei were stained with DAPI. Scale bar, 20 μm. **i** MTT assay performed on PC3 cells after 48 h of treatment with 20 nM docetaxel alone or in presence of 1 mM 3-MA added 1 h before docetaxel treatment. Six independent biological samples for each condition were analysed (*n* = 6), bar graph represents the mean relative cell viability ± SD. Statistical analysis was performed using one-way ANOVA followed by Bonferroni post-test (*p < 0.05). **j** pDsred2-mito fluorescence microscopy analysis shows mitochondrial distribution and localization in PC3 cells treated with 100 mM trehalose, 100 nM rapamycin and/or 20 nM docetaxel (48 h). Scale bar, 20 μm

Docetaxel is a widely used antineoplastic agent in PC^53^. Unfortunately, patients develop docetaxel-resistance afterwards. Multiple molecular mechanisms contribute to this chemoresistance including apoptosis inhibition^8^. We analysed whether docetaxel-induced cell death might be mediated by autophagy. In PC3 cells, docetaxel induced a dose-dependent toxicity from 10 to 100 nM (Fig. 4a). Docetaxel-induced apoptosis was analysed evaluating caspase-3 cleavage. WB analysis showed that docetaxel (20 nM) induced caspase-3 cleavage after 48 and 72 h (Fig. 4b) confirming that, in PC3 cells, docetaxel induces apoptotic cell death. We also analysed whether docetaxel induces autophagy in PC3 cells and whether autophagy modulates docetaxel-induced apoptosis. Notably, docetaxel increased *LC3* expression (Fig. 4c), LC3-II/LC3-I ratio after 48 and 72 h (Fig. 4d), and modified LC3 distribution from diffuse to punctate staining (Fig. 4e). p62 mRNA was increased after 48 and 72 h of docetaxel treatment (Fig. 4f), while no changes were found in p62 protein levels (Fig. 4g), which accumulated into p62 bodies (Fig. 4h). To determine how autophagy mediates docetaxel-induced toxicity, we cotreated PC3 cells with docetaxel and 3-MA and we found that 3-MA has no effect on docetaxel-cytotoxicity (Fig. 4i).

Since in PC3 cells docetaxel toxicity is exerted via apoptosis, we tested how the intrinsic apoptotic pathway is involved by evaluating mitochondrial morphology and the possible modulation by autophagy. Indeed, mitochondria dynamics, fission and fusion, exert paradoxical roles in cell survival and death^54^. Mitochondrial fission generates morphologically and functionally distinct mitochondria and often occurs in the apoptotic event^55^. In PC3 cells, we evaluated whether this is a determinant event in chemo-sensitivity. In live fluorescence microscopy we observed that pDsred2-mito labelled mitochondria underwent fission after docetaxel treatment for 48 h, confirming that this process could mediate docetaxel-induced cell death. Trehalose alone partially induced mitochondrial fission, while docetaxel and trehalose administered together induced mitochondrial aggregation. Rapamycin alone induced mitochondrial fragmentation, which remained unchanged after docetaxel treatment (Fig. 4j).

### Trehalose and rapamycin differently modify mitochondria/autophagosomes colocalization in docetaxel-treated PC3 cells

Since trehalose, rapamycin and docetaxel, either used alone or in combination, induce mitochondrial fission, we tested a possible mitophagy activation by analysing whether autophagosomes colocalize with fragmented mitochondria. In fact, mitophagy, by clearing damaged mitochondria, is critical for cancer cells survival under different stresses^56^, possibly acting by reducing apoptosis^19,57^. IF analysis and confocal microscopy were used to detect mitophagy activation evaluating LC3 or p62 colocalization with MitoTracker Orange-stained mitochondria^58^.

**Fig. 5:**
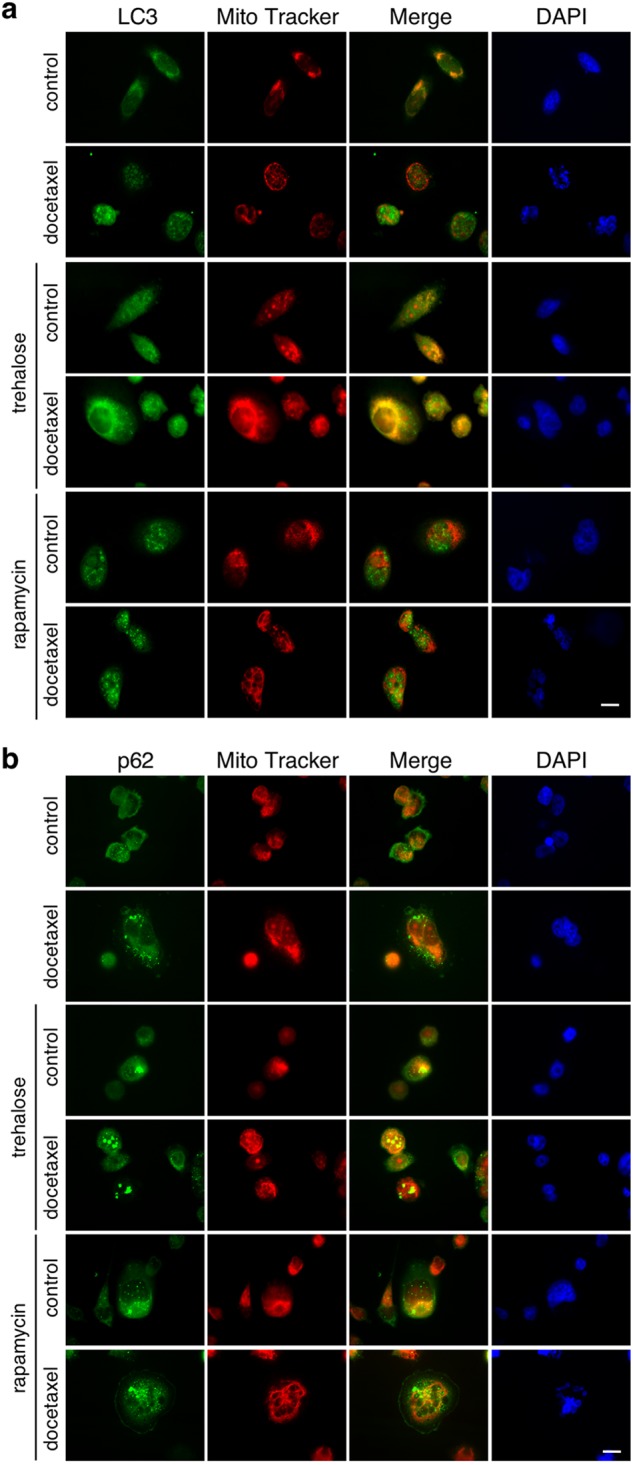
Trehalose and rapamycin differently modify LC3/mitochondria and p62/mitochondria colocalization in docetaxel-treated PC3 cells. **a** PC3 cells were treated for 48 h with 100 mM trehalose or 100 nM rapamycin and/or 20 nM docetaxel. The cells were stained with 250 nM MitoTracker Orange for 30 min to stain the mitochondria and then fixed with paraformaldehyde. IF shows LC3 and mitochondria localization. Nuclei were stained with DAPI. **b** PC3 cells were treated for 48 h with 100 mM trehalose or 100 nM rapamycin and/or 20 nM docetaxel. The cells were stained with 250 nM MitoTracker Orange for 30 min to stain the mitochondria and then fixed with paraformaldehyde. IF shows p62 and mitochondria localization. Nuclei were stained with DAPI. Images were acquired by Zeiss Axiovert 200 microscope equipped with ×63/1.4 objective lens linked to a Coolsnap Es CCD camera (Ropper Scientific-Trenton, NJ, USA), scale bar 20 μm

In PC3 cells a clear colocalization of LC3-labelled autophagosomes and mitochondria was found after trehalose treatment, which was further confirmed by cotreatment with docetaxel. Docetaxel alone, rapamycin and their cotreatments did not induce autophagosomes and mitochondria colocalization, and the two organelles remained confined into different cytoplasm regions. As expected, docetaxel treatment induced nuclear fragmentation, which was not modified by cotreatment with rapamycin, while trehalose completely reverted the docetaxel-induced phenotype at nuclear levels (Fig. 5a and Fig. S2). Thus, trehalose has a potent protective activity against docetaxel-induced apoptosis. p62 was also found colocalized with mitochondria both in trehalose- or trehalose−docetaxel-treated cells. p62/mitochondria colocalization was absent in rapamycin- or docetaxel-treated (alone or in combination) cells (Fig. 5b and Fig. S3). Thus, the apoptotic stress induced by docetaxel alters mitochondria morphology and distribution, but trehalose eliminates damaged mitochondria by mitophagy, while rapamycin induces a nonselective autophagy that does not involve mitophagy.

### Trehalose and rapamycin differently modify mitochondria/lysosomes colocalization in docetaxel-treated PC3 cells

**Fig. 6:**
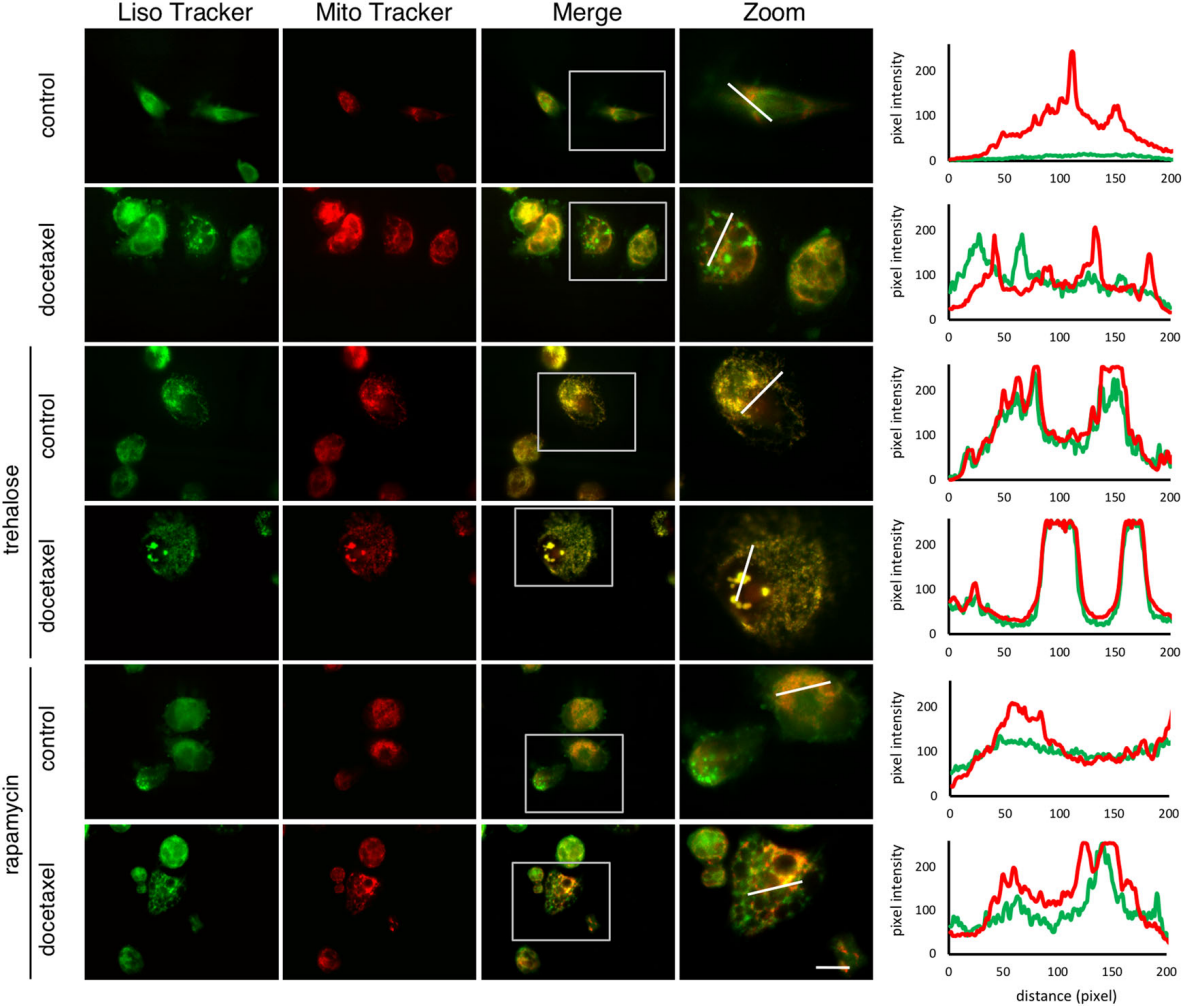
Trehalose and rapamycin differently modify mitochondria/lysosomes colocalization in docetaxel-treated PC3 cells. PC3 cells were treated for 48 h with 100 mM trehalose or 100 nM rapamycin and/or 20 nM docetaxel. The cells were stained with 250 nM MitoTracker Orange for 30 min to stain the mitochondria. Lysosomal staining was performed with 50 nM LysoTracker Green for 45 min. Images were acquired with ×63/1.4 objective lens linked to a Coolsnap Es CCD camera (Ropper Scientific-Trenton, NJ, USA). Scale bar 20 μm. Graphs in the right panel show green and red fluorescence intensity of highlighted dot

To further evaluate these differential effects of trehalose and rapamycin on mitochondria clearance via autophagy, we evaluated mitochondria (stained with MitoTracker Orange) and lysosomes (stained with LysoTracker Green) distribution in PC3 cells and we found that trehalose alone or in the presence of docetaxel induced mitochondria and lysosomes colocalization (Fig. 6), confirming mitophagy activation. Conversely, controls, docetaxel or rapamycin (with or without docetaxel) treated cells had a clear separation between these cytoplasmic organelles. Furthermore, rapamycin-treated cells, with or without docetaxel, showed flattened morphology and large vacuoles typical of a complete autophagic process.

### Trehalose and rapamycin different counteract docetaxel-induced apoptosis in PC3 cells

**Fig. 7:**
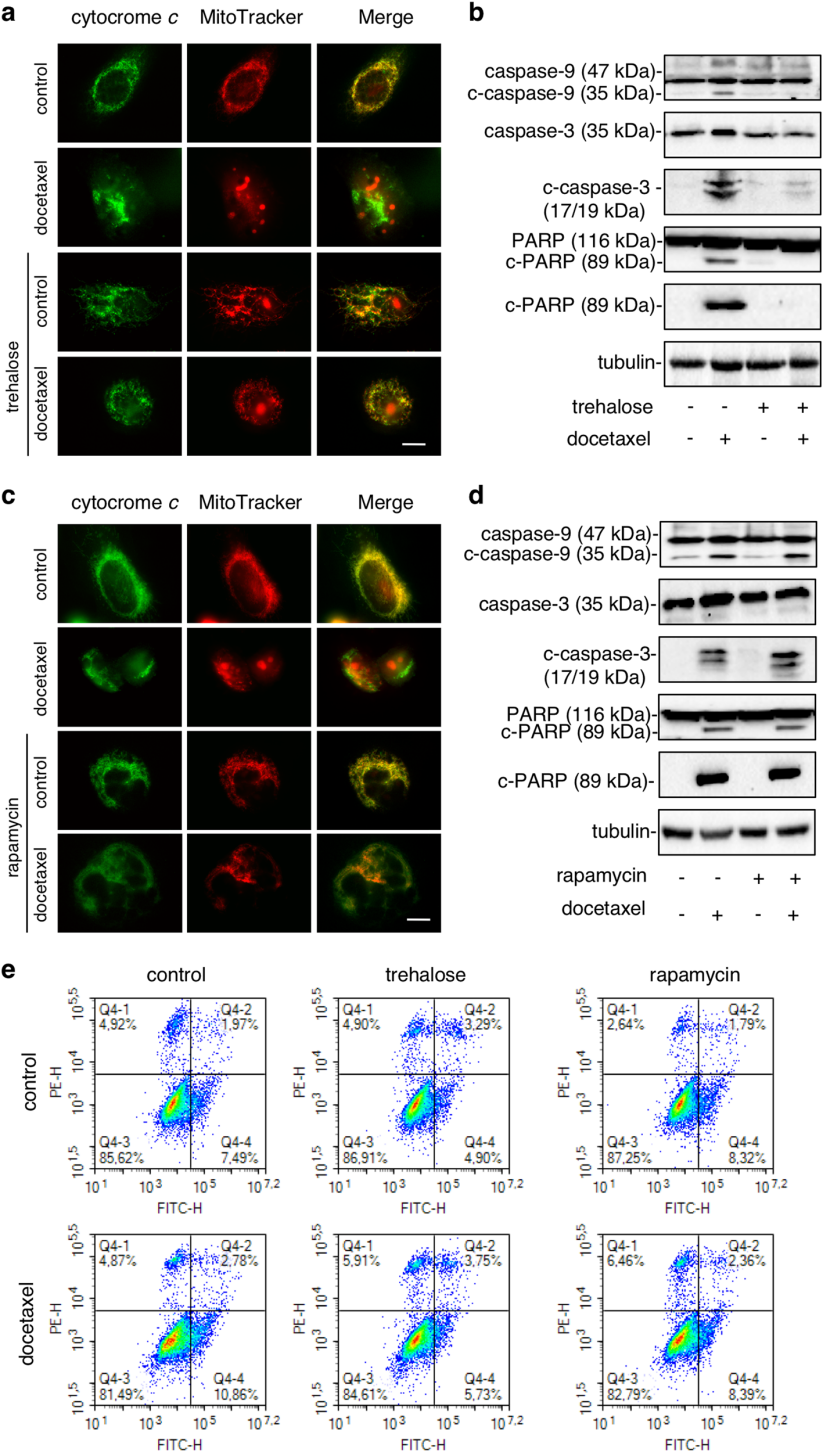
Trehalose and rapamycin differently counteract docetaxel-induced apoptosis in PC3 cells. **a** Mitochondrial localization of cytochrome *c* was evaluated by IF. Cells were treated with 20 nM docetaxel and/or 100 mM trehalose. After 48 h, the cells were incubated for 30 min with 250 nM MitoTracker Orange, fixed and stained with cytochrome *c* antibody followed by FITC secondary antibody. Images were acquired with ×63/1.4 objective lens linked to a Coolsnap Es CCD camera (Ropper Scientific-Trenton, NJ, USA). Scale bar 20 μm. **b** WB shows caspase-9, caspase-3 and PARP and their cleaved active form after 48 h of treatment with 20 nM docetaxel and/or 100 mM trehalose. Each experiment was repeated three times and representative blots are shown. **c** Cells were treated with 20 nM docetaxel and/or 100 nM rapamycin. The mitochondrial localization of cytochrome *c* was evaluated by IF analysis as described in **a**. **d** The cells were treated with 20 nM docetaxel and/or 100 nM rapamycin for 48 h. The analysis of apoptotic-related proteins was conducted as described in **b**. **e** PC3 cells were treated with 20 nM docetaxel, 100 mM trehalose and/or 100 nM rapamycin. After 24 h of the treatment the cells were labelled with Annexin V-FITC and PI. Dot-plots represent flow cytometry analysis of 10,000 events. Experiment was repeated three times and representative plots are shown

To prove that the trehalose-induced mitophagy correlates with apoptotic capability of cancer cells, we analysed the impact of the intrinsic apoptotic pathway and its correlation with mitochondria dynamics, analysing the effect of trehalose and rapamycin on docetaxel-induced cytochrome *c* release, activation of caspase-9, caspase-3 and the cleavage of PARP. Cytochrome *c* colocalization with MitoTracker Orange-stained mitochondria was evaluated by IF microscopy in PC3 cells treated with docetaxel and trehalose alone or in combination. In basal condition, or in cells treated only with trehalose, cytochrome *c* and MitoTracker colocalized docetaxel-induced cytochrome *c* release from mitochondria (Fig. 7a), while trehalose completely reverted the cytochrome *c* release from mitochondria to cytoplasm induced by docetaxel (Fig. 7a). Thus, trehalose likely counteracts docetaxel-induced apoptosis, possibly by promoting the clearance of damaged mitochondria through mitophagy and by increasing resistance to apoptotic death. Analysing caspase-9, caspase-3, PARP and their active cleaved forms (Fig. 7b), we found that docetaxel triggered intrinsic apoptosis pathway via cleavage of caspase-9, 3 and PARP, while trehalose was inactive on these parameters, but fully counteracted the proapoptotic action of docetaxel on all proteins tested. Again, in basal condition, or in cells treated only with rapamycin, cytochrome *c* and MitoTracker colocalized, suggesting that rapamycin is unable to induce cytochrome *c* mitochondrial release, or to activate caspase-9, 3 and PARP and was unable to counteract the docetaxel-induced cytochrome *c* release from mitochondria (Fig. 7c) and the docetaxel ability to trigger apoptosis via caspases (Fig. 7d). The effects of trehalose and rapamycin on the docetaxel-induced cell death were also analysed by PI and annexin V double staining using flow cytometry (Fig. 7e). Docetaxel treatment increased the number of apoptotic PC3 cells by about 40% (from 9.46 to 13.64%) and trehalose cotreatment reduced apoptotic PC3 cells by about 30% (from 13.64 to 9.48%) and drastically reduced early apoptotic PC3 cells (50% from 10.86 to 5.73%). As expected rapamycin cotreatment did not alter apoptotic PC3 cells induced by rapamycin alone (10.75 compared to 10.11) even if it increased necrotic PC3 cells. Moreover, in high-dose docetaxel-treated PC3 cells trehalose reduced late apoptotic cells by about 45% (from 6.15 to 2.75%) (Fig. S4). These results are consistent with previous studies and shows that trehalose counteracts docetaxel-induced apoptosis.

### Opposite role of trehalose and rapamycin on docetaxel-induced cell death in PC3 cells

**Fig. 8:**
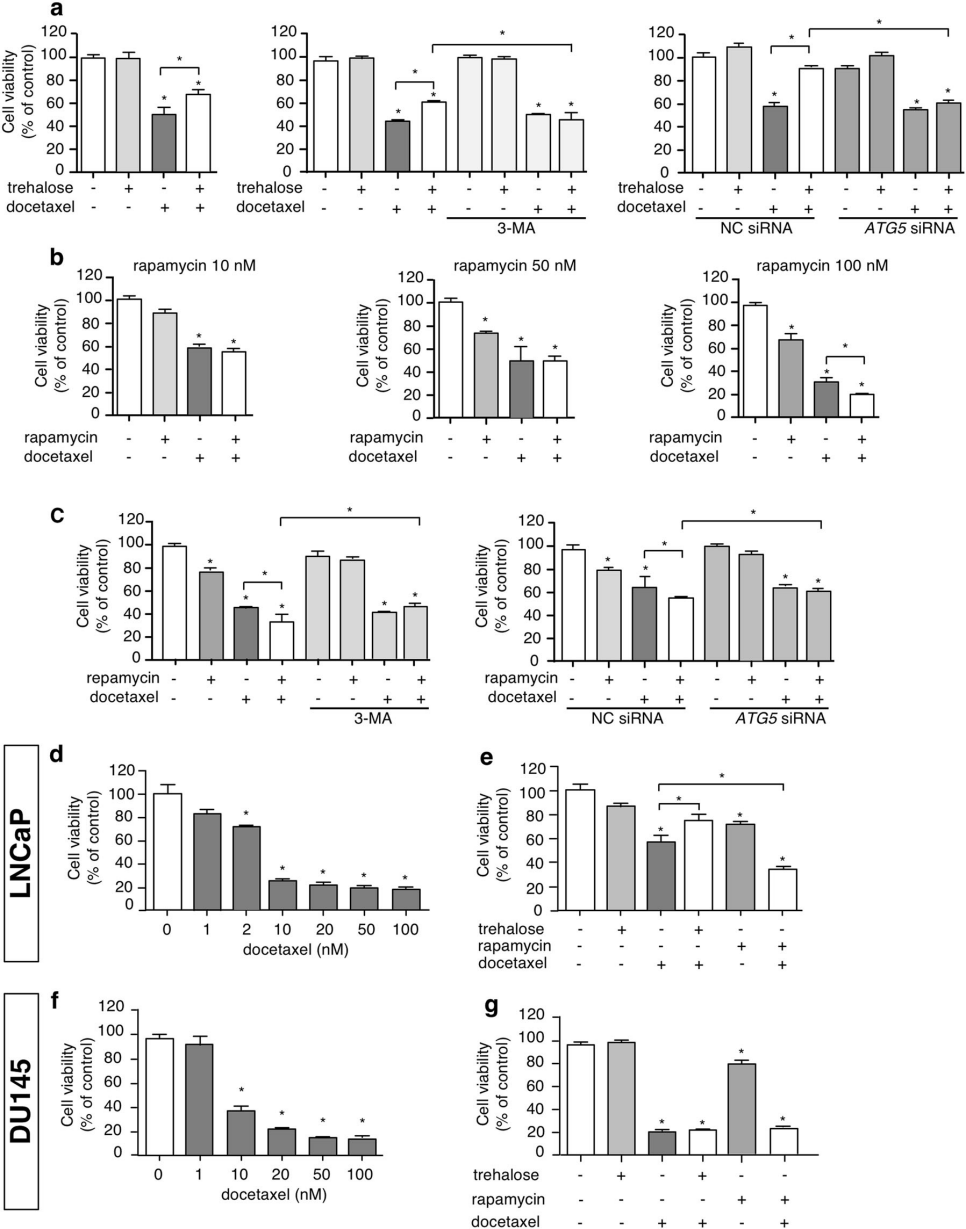
Opposite role of trehalose and rapamycin on docetaxel-induced cell death in PC cells. **a** PC3 cells were treated with 100 mM trehalose and/or 20 nM docetaxel for 48 h. Furthermore, PC3 cells pretreated with or without 1 mM 3-MA for 1 h were exposed to 100 mM trehalose and/or 20 nM docetaxel for additional 48 h. Additionally PC3 cells transfected with 50 nM negative control (NC) or *ATG5* siRNA were exposed to 100 mM trehalose and/or 20 nM docetaxel for additional 48 h. MTT viability assays were performed. Data are mean ± SD of six independent biological samples (*n* = 6). Each experiment was repeated three times. Statistical analysis was performed using one-way ANOVA followed by Bonferroni post-test (*p < 0.05). **b** PC3 cells were treated with different doses of rapamycin (10, 50, 100 nM) in presence of 20 nM docetaxel (48 h). MTT vitality assay was performed. Data are mean of six independent biological samples (*n* = 6). Each experiment was repeated three times. Statistical analysis was performed by one-way ANOVA followed by Bonferroni post-test (*p < 0.05). **c** PC3 cells pretreated with or without 1 mM 3-MA for 1 h were exposed to 100 nM rapamycin and/or 20 nM docetaxel for additional 48 h. Additionally PC3 cells transfected with 50 nM negative control (NC) or *ATG5* siRNA were exposed to 100 nM rapamycin and/or 20 nM docetaxel for additional 48 h. MTT viability assays were performed. Data are mean ± SD of six independent biological samples (*n* = 6). Each experiment was repeated three times. Statistical analysis was performed using one-way ANOVA followed by Bonferroni post-test (*p < 0.05). **d** LNCaP cells were treated with 1, 2, 10, 20, 50, 100 nM docetaxel. MTT assays were performed. Data are mean ± SD of six independent biological samples (*n* = 6). Each experiment was repeated three times. Statistical analysis of data was performed by Dunnet test (*p < 0.05). **e** LNCaP cells were treated with trehalose 100 mM or rapamycin 100 nM in combination with 2 nM docetaxel for 48 h and one-way ANOVA followed by Bonferroni post-test for drugs combination experiments (*p < 0.05). **f** DU145 cells were treated with 1, 10, 20, 50, 100 nM docetaxel. MTT assays were performed. Data are mean ± SD of six independent biological samples (*n* = 6). Each experiment was repeated three times. Statistical analysis of data was performed by Dunnet test (*p < 0.05). **g** DU145 cells were treated with trehalose 100 mM or rapamycin 100 nM in combination with 20 nM docetaxel for 48 h and one-way ANOVA followed by Bonferroni post-test for drugs combination experiments (*p < 0.05)

Since trehalose and rapamycin exert different role on the intrinsic apoptotic pathway triggered by docetaxel, we evaluated whether these autophagy inducers have a different effect on docetaxel-induced cytotoxicity. We found that trehalose treatment partially reduced docetaxel-induced cell death in PC3 cells, but this protective effect was abolished by 3-MA, as well as by the silencing of the *ATG5* gene, which is involved in autophagy activation (Fig. 8a). Rapamycin added at doses ranging from 10 to 100 nM to docetaxel-treated cells was found active to significantly reduce cell viability but only at the highest dose (Fig. 8b) when compared to docetaxel-treated cells. Autophagy inhibition via 3-MA, or genetically by *ATG5* downregulation with a specific siRNA, abrogated the effect of rapamycin and the adverse effects of the combined treatment with rapamycin and docetaxel on cell viability (Fig. 8c). Thus, trehalose-induced mitophagy exerts a cytoprotective effect against chemotherapy, while rapamycin-induced autophagy activates a type of caspase-independent cell death, which synergizes with the apoptotic death induced by docetaxel.

Finally, we tested whether docetaxel, trehalose or rapamycin alone or in combination modify the same parameters in LNCaP and DU145 cells. In LNCaP cells, we observed the same results obtained in PC3 cells (Fig. 8d, e). Conversely, in DU145 cells, the cytotoxicity of docetaxel was not reverted by trehalose. Neither rapamycin increased docetaxel cytotoxic (Fig. 8f, g).

A rescue experiment was performed in DU145 cells in order to evaluate whether these cells may become sensitive to trehalose when autophagy is restored. We thus overexpressed the ATG5 protein in DU145 cells (which are characterized by the absence of this proautophagic factor) and found that trehalose significantly reduced the cytotoxic effect of docetaxel in these cells (Fig. S5). This confirms that autophagy mediates the action of trehalose even in DU145 cells when this pathway is “re-activated”.

## Discussion

Development of chemoresistance is one of the major problem in cancer therapy, in which autophagy may play a role. It is still controversial whether autophagy kills cancer cells or sustains their survival under stressful conditions as chemotherapy or radiation therapy. Many studies argue that autophagy is implicated in cancer cells resistance to chemotherapy and thus autophagy inhibition could improve the anticancer outcome by resensitizing cancer cells to chemotherapy^17,18,59^. Nevertheless, other reports suggest that autophagy enhances the effect of chemotherapy and radiotherapy inducing cell death^60,61^.

Here, we determined if different autophagy activators, trehalose and rapamycin, modulate docetaxel-induced toxicity in CRPC cell lines. We demonstrated that in PC3 cells trehalose induces autophagy by activating LC3 and p62 autophagy markers. Notably, LC3 and p62 degradation were lower than their synthesis. In PC3 cells, also rapamycin induced *LC3* RNA and protein levels, but puncta induction was evident after autophagy flux blockage. Rapamycin enhanced *p62* expression, but reduced p62 protein levels, a phenomenon associated to an active autophagy. Therefore, rapamycin induces autophagy in PC3 cells as previously shown^50–52,62^.

Conversely, while the effects of trehalose and rapamycin were replicated in LNCaP cells, the inducers did not activate autophagy in DU145 cells, which are refractory to autophagy activation because of the lack of full-length *ATG5*^45^. However, when autophagy was “re-activated” by transfecting the ATG5 protein, we found that these cells became sensitive to the trehalose effects on docetaxel-induced cell death.

In PC3 cells docetaxel activates caspase-3 cleavage, suggesting that apoptosis is centrally involved in docetaxel-induced toxicity in PC3 cells^63,64^. The role of autophagy in this context is unclear. Our studies demonstrate that docetaxel induces autophagy according to Zhang et al.^65^, but the inhibition of this process does not alter the sensitivity to docetaxel, in line with studies showing that autophagy does not mediate docetaxel toxicity or resistance^66^. On the other hand our results diverge from a previous report suggesting that 3-MA alters docetaxel-induced toxicity^63^, then this aspect is still controversial.

We also found a direct involvement of the mitochondrial quality control system. Mitochondria are crucial to modulate the cross-talk between autophagy and apoptosis, and regulate cell death or survival decision^67,68^. Studying mitochondria distribution we demonstrated that docetaxel, trehalose and rapamycin induce mitochondria fission, which is mediated by several biological phenomena, including apoptosis and autophagy^69,70^. Here, mitochondrial fission correlated with the apoptosis activation by docetaxel and with autophagy activation by trehalose and rapamycin. The combined treatments of docetaxel with the autophagy inducers demonstrated a clear different spatial redistribution of mitochondria: the combination of trehalose and docetaxel determined the perinuclear mitochondrial aggregation, the combination of rapamycin with docetaxel resulted in a widespread cytoplasmic distribution of mitochondria.

In mammalian cancer cells, these processes are differentially associated to mitochondrial turnover. Indeed, mitochondria damaged by different type of stresses undergo a selective autophagy, named mitophagy^56^, in which membrane potential loss induces mitochondria ubiquitination and p62 recruitment for their insertion into autophagosomes and perinuclear region localization into mito-aggresomes^71,72^. This step precedes their lysosomal degradation. In our experiments, trehalose and docetaxel administered together trigger the initial phases of mitophagy, by inducing a marked relocalization of mitochondria around nuclear envelope, generating mito-aggresomes-like structures. These structures are absent in rapamycin- and docetaxel-treated cells. Mitophagy induction after trehalose and docetaxel exposure is also corroborated by the mitochondria colocalization with LC3- and p62-positive autophagosomes in docetaxel-treated cells. Mitochondria accumulation into autophagosomes was not induced by rapamycin (even in combination with docetaxel), while trehalose alone increased autophago-lysosomes formation causing aggregated mitochondria accumulation into lysosomes. Thus, trehalose induces mitophagy, while rapamycin only activated autophagy with large cytoplasmic vacuoles formation, but not mitophagy. Interestingly, trehalose does not induce apoptosis *per se*, but it counteracts docetaxel-triggered apoptosis, also enhancing the removal of damaged mitochondria by mitophagy. This maintains the pool of mitochondria poorly sensitive to apoptotic stimuli, since mitochondria accumulated into autophago-lysosomes cannot release cytochrome *c* to trigger apoptosis.

Our data parallel previous studies on neurodegenerative diseases, where trehalose induces mitophagy in tau pathology preventing cytochrome *c* release from damaged mitochondria and exerting a protective antiapoptotic role against the disease^27^. This may be viewed as *a Janus* effect of trehalose in human diseases. Indeed, for cancer treatment, this effect represents the other face of the coin, since trehalose protection against cytotoxic stresses induced by chemotherapy attenuated its efficiency, as demonstrated by the reduction of docetaxel-induced cytotoxicity in PC3 cells exposed to trehalose. Notably, rapamycin did not activate apoptosis *per se* and did not modify the proapoptotic activity of docetaxel, but potentiated the anticancer effects induced by docetaxel on PC3 cells. Other studies showed that rapamycin enhances docetaxel^40^ and cisplatin^41^ cytotoxicity in PC3 cells.

Our observations explain data collected in melanoma cells, in which trehalose counteracts cisplatin-mediated apoptosis, whereas the CCI-779 rapamycin analogue is ineffective^73^.

Collectively, the results obtained lead to hypothize that trehalose-induced mitophagy represents a crucial cellular survival response involved in chemotherapy resistance. Alternatively, activation of autophagy mediated by rapamycin is a phenomenon that causes cell death and enhances the effect of chemotherapy (Fig. S6).

In conclusion, our findings clarify that mitophagy is a key mechanism in docetaxel-resistance in CRPC and focus that the molecular mechanisms of autophagy activation are crucial for the therapeutic use of combination therapies.

## Materials and methods

### Antibodies and reagents

Rabbit anti-LC3 (L8918), mouse anti-alpha-tubulin (T6199) and mouse anti-FLAG^®^ clone M2 (F1804) were purchased from Sigma-Aldrich (St. Louis, MO, USA). Rabbit anti-SQSTM1/p62 (PA5-20839) was from ThermoFisher Scientific (Waltham, MA, USA). Rabbit anti-procaspase-3 (#9665), rabbit anti-cleaved-caspase-3 (Asp-175, clone 5A1E) (#9664), mouse anti-procaspase-9 (#9508), rabbit anti-PARP (#9542), rabbit anti-cleaved-PARP (#5625), rabbit anti-Atg5 (#12964) were from Cell Signaling (Danvers, MA, USA). Mouse anti-cytochrome *c* (7H8) (sc-13560) was from Santa Cruz Biotechnology (Santa Cruz, CA, USA). Mouse anti-GFP (Ab1218) was from Abcam (Cambridge, UK). Rabbit and mouse horseradish-peroxidase-conjugated secondary antibody were from Cell Signaling. FITC-conjugated secondary antibody Alexa Fluor 488, MitoTracker Orange CMTM Ros (mitochondrial selective dye) (M7510) and the LysoTracker Green DND-26 (lysosomal selective dye) (L7526) were acquired from Molecular Probes (ThermoFisher Scientific). Trehalose (T0167), rapamycin (R0395), chloroquine (CQ) (C6628), ammonium chloride (NH_4_Cl) and docetaxel were purchased from Sigma-Aldrich. 3-MA (S2767) was obtained from Selleckchem (Munich, Germany).

### Plasmids and siRNA

pcDNA5/TO-polyQ plasmid was used to express polyQ containing protein, it was constructed by cloning the sequence of N-term polyQ tract of Huntingtin into *Hin*dIII and *Not*I sites of pcDNA5/TO (Life Technologies, V103320). This sequence contains an N-term FLAG tag.

pCI-neo-hApg5-HA was a gift from Noboru Mizushima (Addgene plasmid # 22948). It was used to express wild-type *ATG5* gene in DU145 cells^74^.

The pEGFPN1 (Clontech, U55762) plasmid was used to evaluate transfection efficiency in experiments involving transient transfections.

To silence endogenous *ATG5* expression we used negative control (NC) siRNA (#6568) and *ATG5* siRNA I (#6345) obtained from Cell Signaling (#6345).

### Cell culture and treatments

Human PC cell lines, LNCaP, PC3 and DU145, were purchased from the American Type Culture Collection (ATCC, Manassas, VA, USA). The cells were authenticated using Short Tandem Repeat analysis as described in ANSI Standard (ASN-0002) by ATCC Standards Development Organization (SDO). LNCaP, PC3 and DU145 cells  were maintained in RPMI-1640 medium (EuroClone, Milano, Italy) supplemented with 10% (PC3) and 5% (LNCaP and DU145) fetal bovine serum (FBS) (Gibco, ThermoFisher Scientific), glutamine (1 mmol/l) and antibiotics (100 IU/ml penicillin G) and cultured at 37 °C in humidified atmosphere of 5% CO_2_.

### Cell viability assay

LNCaP, PC3 and DU145 cells were plated at the density of 3×10^4^ cells/well in 24-well plates. After 48 h cells were treated with different drugs. At the end of the treatment the medium was changed with MTT (3-(4,5 dimethylthiazol-2-yl)-2,5-diphenyl tetrazolium bromide) solution (0.5 mg/ml) in RPMI without phenol red and FBS and the cells were incubated for 30–45 min. The precipitate formazan was dissolved with isopropanol. Absorbance at 550 nm was measured by EnSpire Multimode Plate reader (Perkin Elmer, Milano, Italy).

### mRNA expression analysis

PC3 cells were plated at 25×10^4^ cells/well in six-well multiwell plates, allowed to growth for 48 h and then treated with 100 mM trehalose or 100 nM rapamycin. 48 or 72 h after treatments, cells were harvested and centrifuged for 5 min at 100 × *g* at 4 °C; the pellets were resuspended in 300 μl of TRI Reagent (#T9424; Sigma-Aldrich) and total RNA isolated according to the manufacturer’s instructions. RNA quantification was carried out by absorbance at 260 nm. One microgram of total RNA was treated with DNAse I (AMPD1; Sigma-Aldrich) and reverse transcribed into cDNA using the High-Capacity cDNA Archive Kit (4368813; Life Technologies) according to the manufacturer’s protocol. All primers for real-time PCR were designed using the program Primer 3. The primers were synthesized by MWG Biotech (Ebersberg, Germany) with the following sequence: hMAP-LC3-B 5′-CAG CAT CCA ACC AAA ATC CC-3′ (forward), 5′-GTT GAC ATG GTC AGG TAC AAG-3′ (reverse); hSQSTM1/p62 5′-CCA GAG AGT TCC AGC ACA GA-3′ (forward), 5′-CCG ACT CCA TCT GTT CCT CA-3′ (reverse); hRplP0: 5′-GTG GGA GCA GAC AAT GTG GG-3′ (forward), 5′-TGC GCA TCA TGG TGT TCT TG-3′ (reverse).

The evaluated efficiency of each set of primers was close to 100% for both target and reference gene. Real-time PCR was performed using the CFX 96 Real-Time System (Bio-Rad) in a 10 μl total volume, using the iTaq SYBR Green Supermix (Bio-Rad), and with 500 nM primers. PCR cycling conditions were as follows: 94 °C for 10 min, 40 cycles at 94 °C for 15 s and 60 °C for 1 min. Melting curve analysis was performed at the end of each PCR assay as a control for specificity. Data were analysed and expressed as previously described^75–77^. Values were normalized to those of RplP0. All statistics were performed with ∆C_t_ values.

### Western blotting

For WB studies LNCaP, PC3 and DU145 cells were seeded at 3×10^5^ cells/plate in 10 cm dishes. After 48 h, cells were treated with drugs and at the end of experiments they were washed with PBS and lysed with RIPA buffer (0.05 mol/l Tris HCl pH 7.7, 0.15 mol/l NaCl, 0.8% SDS, 10 mmol/l EDTA, 100 μM/l NaVO_4_, 50 mmol/l NaF, 0.3 mmol/l PMSF, 5 mmol/l iodoacetic acid) containing leupeptin (50 μg/ml), aprotinin (5 μl/ml) and pepstatin (50 μg/ml). Protein concentration was determined using BCA protein assay kit (ThermoFisher Scientific). 15–35 μg of proteins were separated through SDS gel electrophoresis and transferred to PVDF (for LC3 analysis) or nitrocellulose membranes. After blocking, membranes were incubated at 4 °C overnight using the following antibodies: anti-LC3 (dilution 1: 2500), anti-SQSTM1/p62 (dilution 1:3000), anti-ATG5 (dilution 1:1000), anti-procaspase-3 (dilution 1:1000), anti-cleaved-caspase-3 (dilution 1:500), anti-procaspase-9 (dilution 1:1000), anti-PARP (dilution 1:1000), anti-cleaved-PARP (dilution 1:1000), anti-GFP (dilution 1:2000), anti-FLAG^®^ clone M2 (dilution 1:1000). Peroxidase-conjugated secondary anti-rabbit or anti-mouse antibodies were used for 1 h at room temperature. The membranes were processed using enhanced chemiluminescence kit ECL Prime Western Blotting Detection Reagent (GE Healthcare Life Sciences Italia, Milano, Italy).

In each WB experiment tubulin expression (dilution 1:2000) was evaluated as a loading control.

### Knockdown of *ATG5* gene in PC3 cells

PC3 cells were seeded in six-well multiwell plates (for WB) and 24-well multiwell plates (for MTT assay) for 24 h. Cells were transfected with 50 nM *ATG5* siRNA or negative control siRNA by Lipofectamine 3000 (Life Technologies−ThermoFischer Scientific) according to the manufacturer's instructions. After 24 h medium was replaced with specific treatments.

### Transient expression of wild-type *ATG5* gene in DU145 cells

DU145 cells were seeded in six-well multiwell plates (for WB) and 24-well multiwell plates (for MTT assay) for 24 h. Cells were transfected with 1 μg of pCI-neo-hApg5-HA by Lipofectamine 3000 (Life Technologies−ThermoFischer Scientific) according to the manufacturer’s instructions. After 5 h medium was replaced with specific treatments.

### Filter retardation assay (FRA)

PC3 cells were plated in six-well multiwell plates at 100 000 cell/well. Twenty-four hours after plating, cells were transfected with 0.5 μg of pcDNA5/TO-polyQ by Lipofectamine 3000 (Life Technologies−ThermoFischer Scientific) according to the manufacturer’s instructions. After 5 h medium was replaced with trehalose 100 mM. Fifty-three hours after transfection, cells were harvested and centrifuged 5 min at 100 × *g* at 4 °C; the cell pellets were resuspended in PBS (Sigma-Aldrich, P4417) added of the protease inhibitor cocktail (Sigma-Aldrich, P8340) and homogenized using slight sonication to lyse cells and nuclei. Total proteins were determined with the bicinchoninic acid method (BCA assay; Euroclone, EMP014500). FRA was performed using a Bio-Dot SF Microfiltration Apparatus (Bio-Rad). Eight micrograms of the total proteins or 1.5 mg of the total protein were filtered through a 0.2-μm cellulose acetate membrane (Whatman, 100404180). Slot-blots were probed as described for WB with anti-FLAG^®^ clone M2.

A ChemiDoc XRS System (Bio-Rad, Hercules, California, USA) was used for the image acquisition of FRA. Optical density of samples assayed with FRA was detected and analysed using the Image Lab software (Bio-Rad).

Statistical analyses have been performed using the relative optical densities defined as the ratio between optical densities of each independent biological sample (*n* = 3) and the mean optical density of control samples.

### Immunofluorescence and confocal microscopy

For immunofluorescence studies the cells were seeded at 3×10^4^ cells/well on polilysine-coated coverslips. After 48 h, cells were treated and fixed with 3% paraformaldeyde/2% sucrose. Cells were washed with PBS and permeabilized with 0.1% Triton X-100 in PBS for 20 min followed by incubation in blocking solution (1% horse serum in PBS) for 1 h.

Cells were incubated with the following antibodies diluted in PBS with 3% BSA overnight at 4 °C: anti-LC3 (dilution 1:1000), anti-SQSTM1/p62 (dilution 1:1000), anti-cytochrome *c* (dilution 1:200). The cells were washed with PBS and incubated with secondary antibodies Alexa-488 for 1 h at room temperature. Nuclei were stained with DAPI (dilution 1:10 000). Labelled cells were examined under Zeiss Axiovert 200 microscope (Zeiss, Oberkochen, Germany) with ×63/1.4 objective lens linked to a Coolsnap Es CCD camera (Ropper Scientific-Trenton, NJ, USA). Confocal microscopy images were acquired with LSM510 Meta system confocal microscope (Zeiss) and processed with the Aim 4.2 software (Zeiss).

### Live mitochondrial morphology

PC3 cells were grown on coated-polilysine LabTeck Chamber Slides (Nalgene Nunc). Mitochondria were detected by transfecting cells with pDsred2-mito Vector (Clontech-Takara Bio USA). Transfection was performed with Lipofectamine 3000 (Life Technologies−ThermoFischer Scientific) according to the manufacturer’s instructions. After 5 h, the cells treated with docetaxel, trehalose or rapamycin alone or in combination for 48 h. Labelled cells were examined by live microscopy using Zeiss Axiovert 200 microscope (Zeiss, Oberkochen, Germany) with ×63/1.4 objective lens linked to a Coolsnap Es CCD camera (Ropper Scientific-Trenton, NJ, USA).

### Mitochondrial and lysosomal staining

PC3 cells were plated at 3×10^4^ cells/well on coated-polilysine coverslips. After the treatment, they were stained with 250 nM MitoTracker Orange for 30 min to stain the mitochondria and with 50 nM LysoTracker Green for 45 min to stain the lysosomes. Then, the cells were washed with PBS, fixed with paraformaldehyde and analysed using Zeiss Axiovert 200 microscope or confocal microscope.

### Apoptosis analysis by flow cytometry

Apoptotic cell death was evaluated by flow cytometry Annexin V/PI double staining. Cells were plated at 2×10^5^ cells/plate in 6 cm dishes and treated with trehalose, rapamycin and/or docetaxel for 24 h. After treatment, cells were harvested by trypsin treatment, and resuspended in binding buffer and stained with Annexin V and PI according to the manufacturer’s instructions (BMS500FI, eBioscience). Immediate analysis of 10 000 event/sample was performed using flow cytometry to observe fluorescence. Flow cytometry was conducted using a NovoCyte 3000 (Acea Bioscience, Inc.). Data were analysed using NovoExpress (Acea Bioscience, Inc.).

### Statistical analysis

All experiments were performed three times and the results were analysed by unpaired Student’s *t* test or by one-way analysis of variance ANOVA, followed by Dunnet or Bonferroni post-test using the PRISM software (GraphPad Software, La Jolla, CA, USA).

## Electronic supplementary material

Supplementary Figures

Legends to Supplementary figures
