# Supplementary material for: Dual role of autophagy on docetaxel-sensitivity in prostate cancer cells
Source: Cell Death Dis. 2018 Aug 30;9(9):889. doi: 10.1038/s41419-018-0866-5 (PMC6117300; doi:10.1038/s41419-018-0866-5)
Supplement: Supplementary file 1 — Supplementary Figures [file 41419_2018_866_MOESM1_ESM.pptx]

## Slide 1
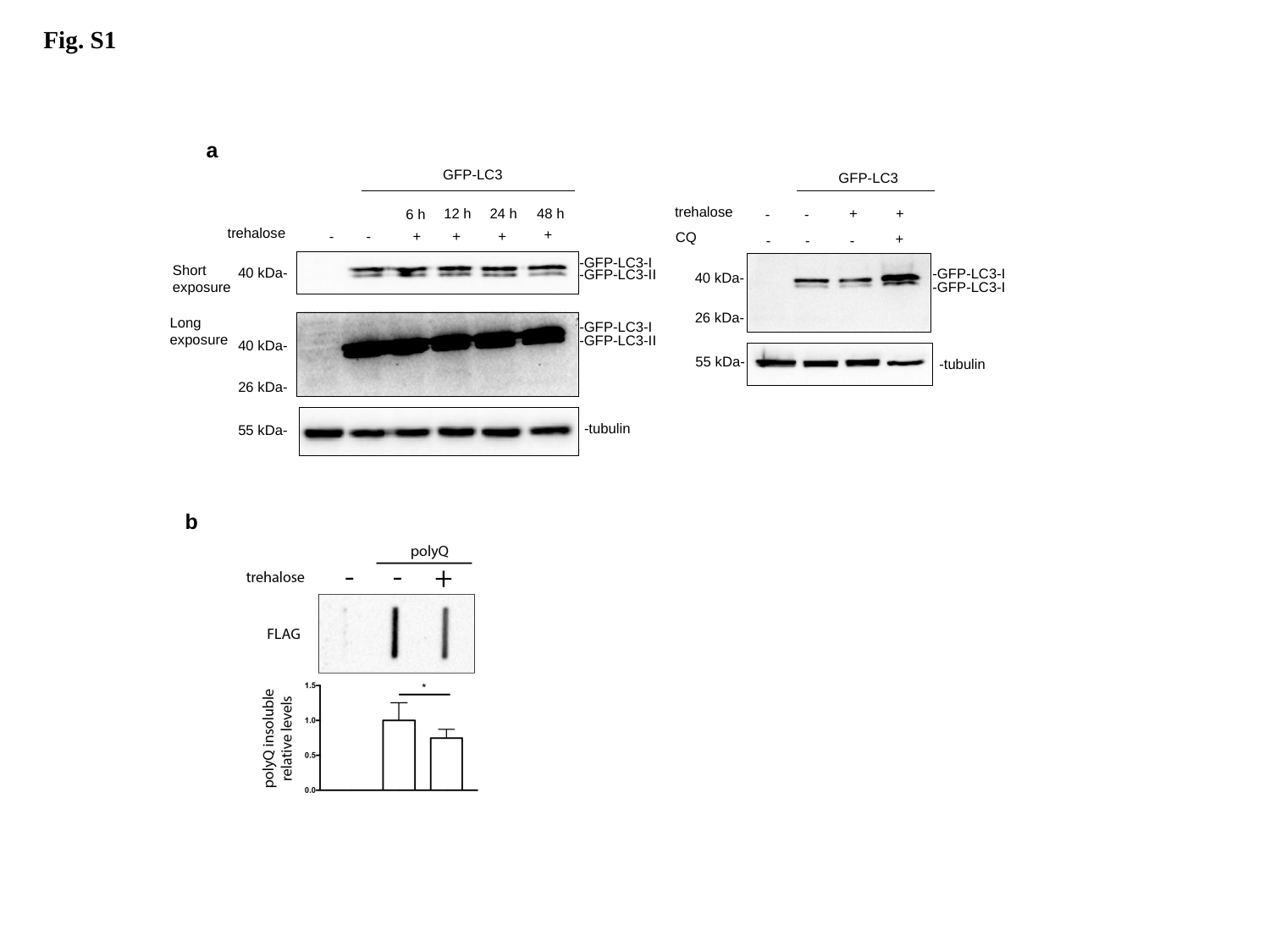

Fig. S1
a
GFP-LC3
GFP-LC3
trehalose
+
+
-
-
CQ
+
-
-
-
-GFP-LC3-I
40 kDa-
-GFP-LC3-I
26 kDa-
-tubulin
12 h
24 h
48 h
6 h
trehalose
+
+
-
-
+
+
-GFP-LC3-I
Short
exposure
40 kDa-
-GFP-LC3-II
Long
exposure
-GFP-LC3-I
-GFP-LC3-II
40 kDa-
55 kDa-
26 kDa-
-tubulin
55 kDa-
b

## Slide 2
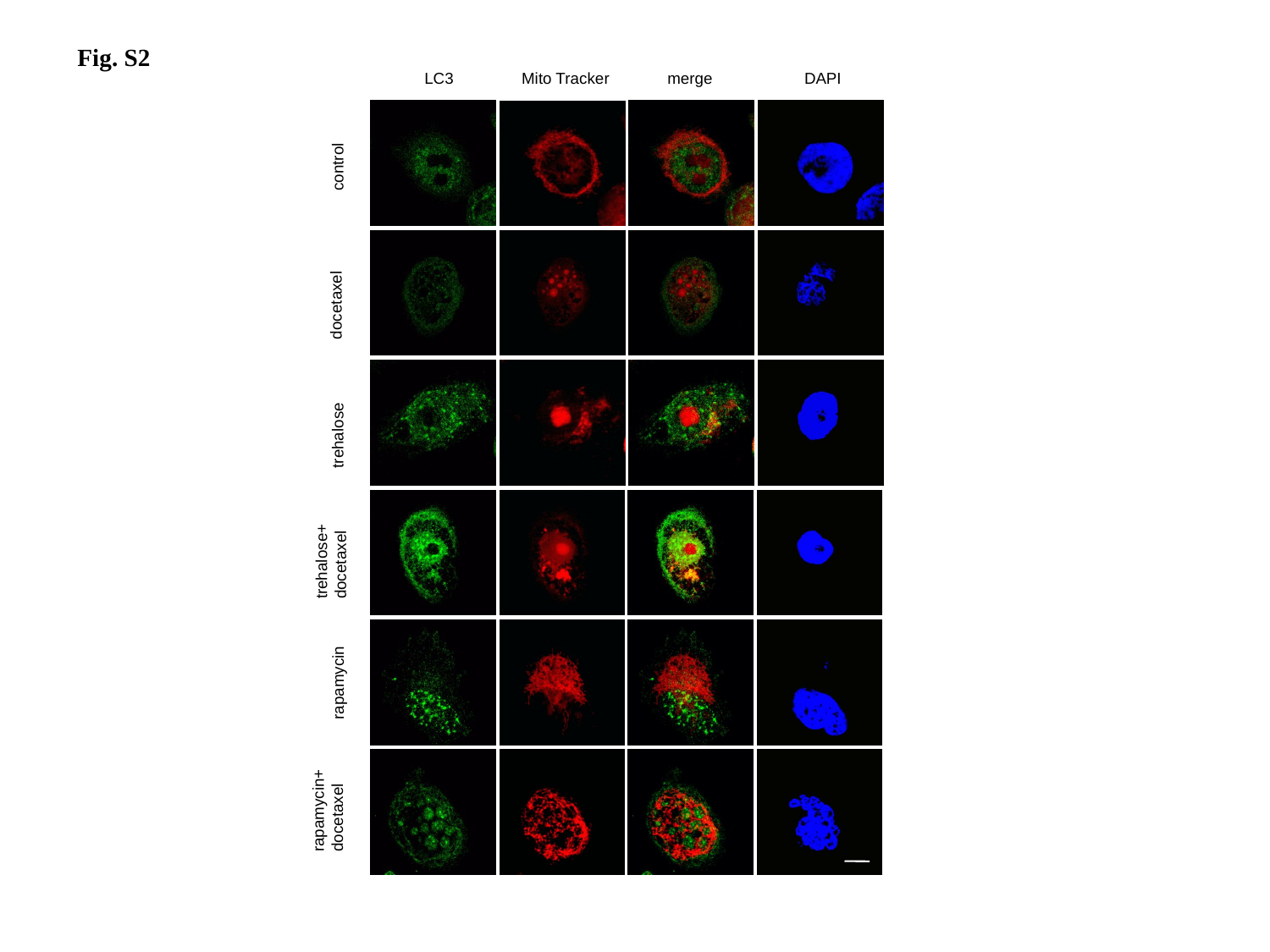

Fig. S2
LC3
Mito Tracker
merge
DAPI
control
docetaxel
trehalose
trehalose+
docetaxel
rapamycin
rapamycin+
docetaxel

## Slide 3
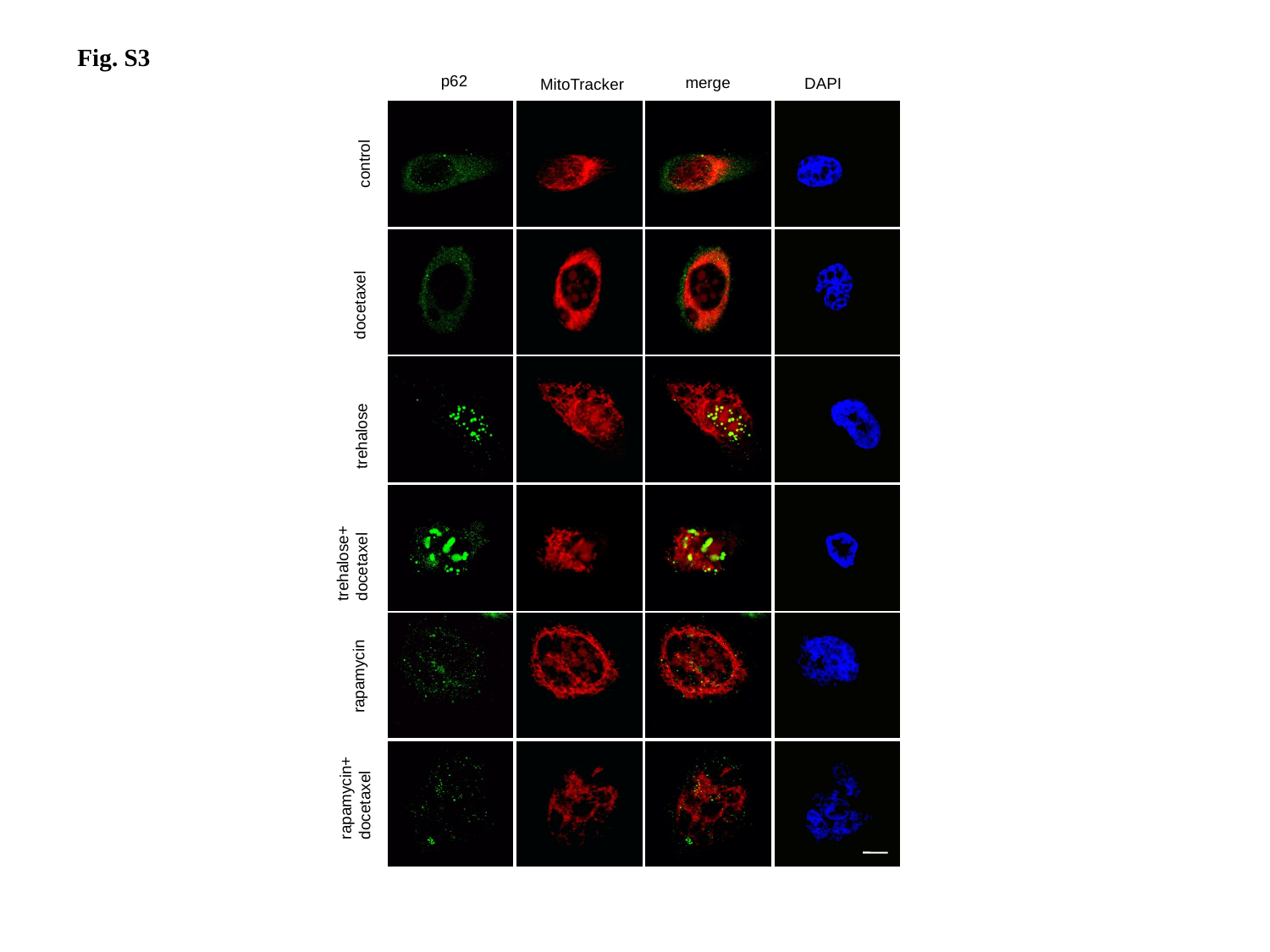

Fig. S3
p62
merge
DAPI
MitoTracker
control
docetaxel
trehalose
trehalose+
docetaxel
rapamycin
rapamycin+
docetaxel
TOGLIERE

## Slide 4
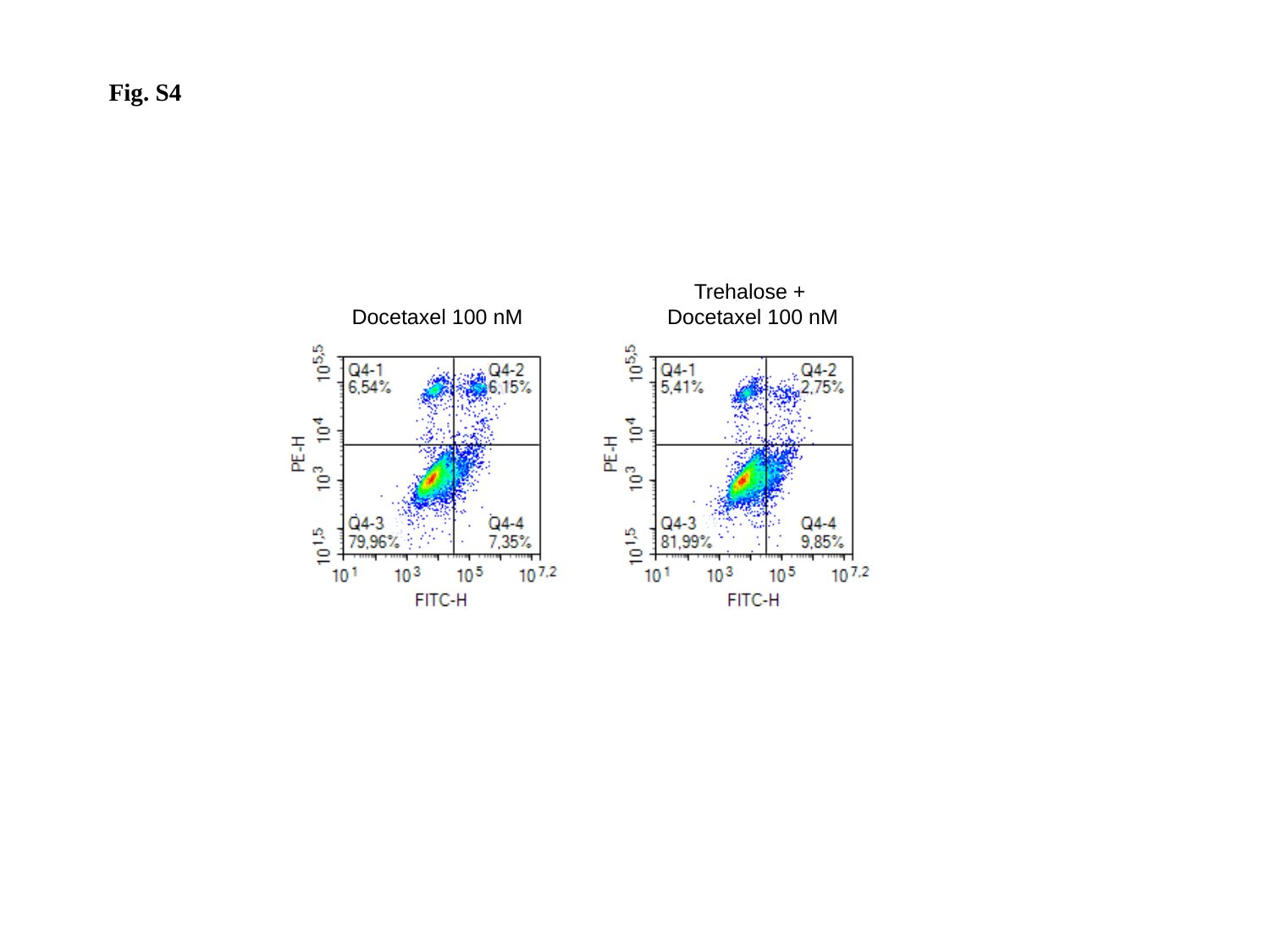

Fig. S4
Trehalose +
Docetaxel 100 nM
Docetaxel 100 nM

## Slide 5
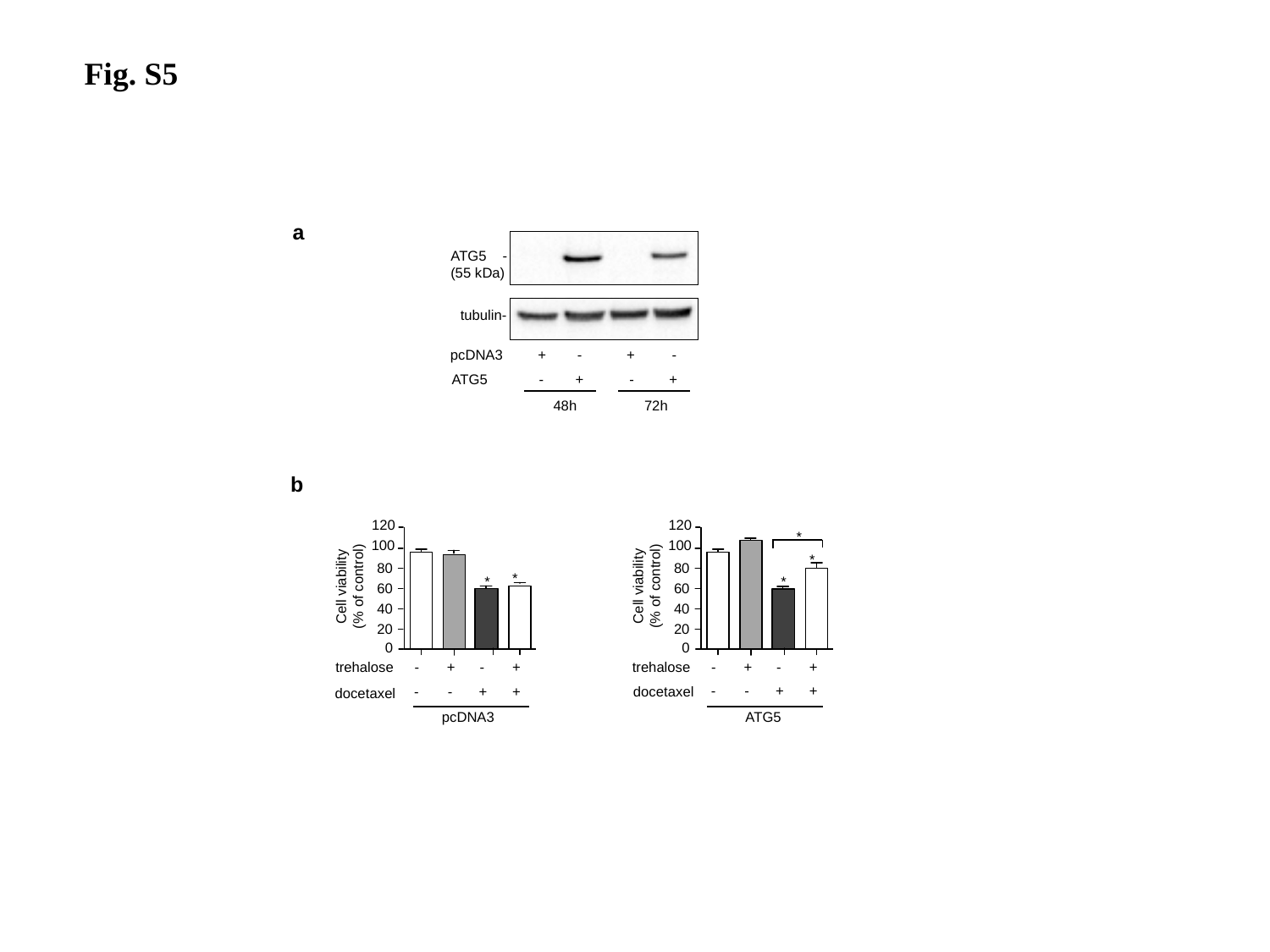

Fig. S5
a
ATG5 -
(55 kDa)
tubulin-
+
-
+
-
pcDNA3
ATG5
-
+
-
+
48h
72h
b
120
100
80
*
Cell viability
(% of control)
*
60
40
20
0
-
+
-
+
trehalose
-
-
+
+
docetaxel
120
100
*
80
Cell viability
(% of control)
*
60
40
20
0
-
+
-
+
trehalose
-
-
+
+
docetaxel
*
pcDNA3
ATG5

## Slide 6
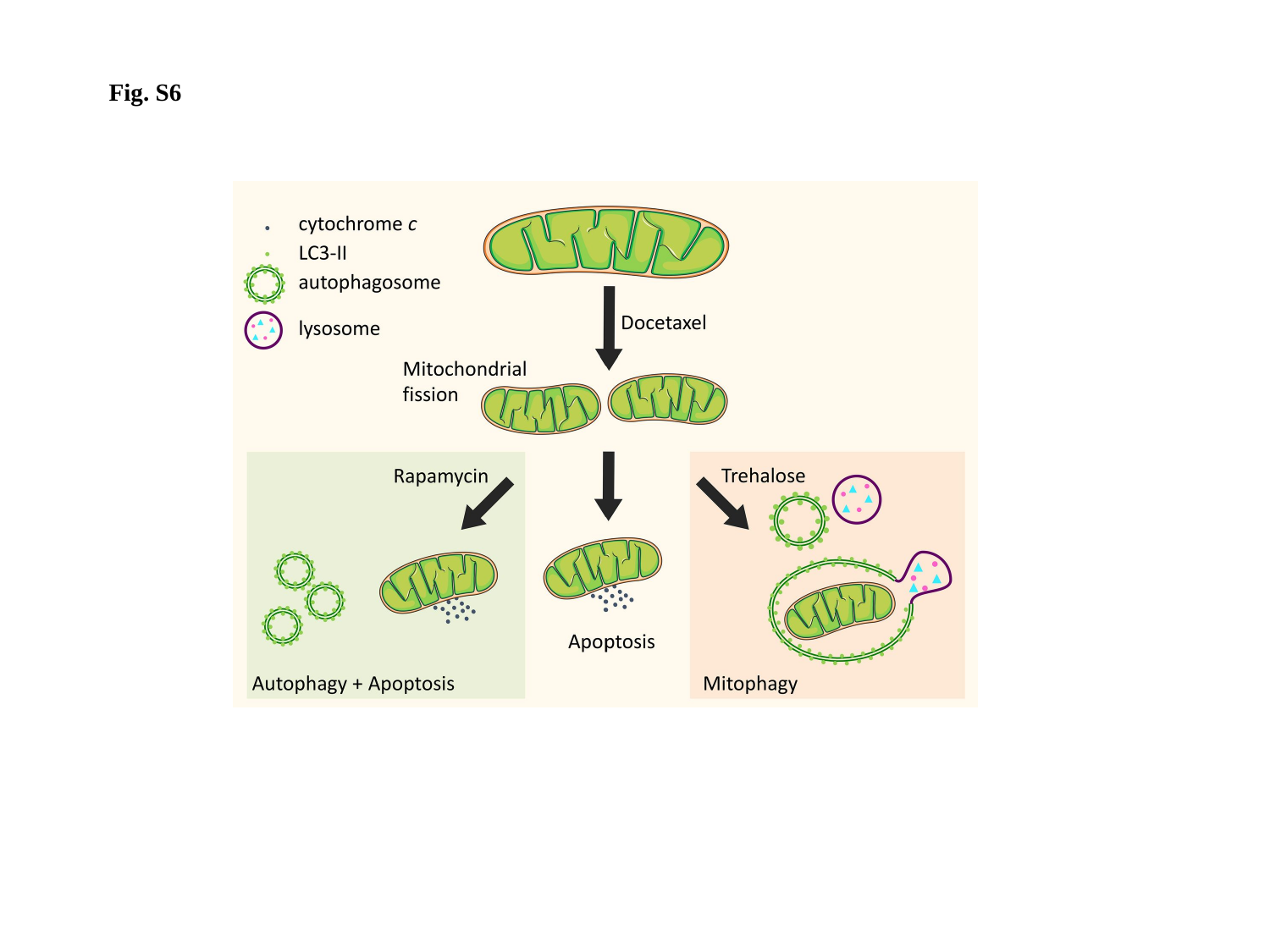

Fig. S6
