# Supplementary material for: Dual role of autophagy on docetaxel-sensitivity in prostate cancer cells
Source: Cell Death Dis. 2018 Aug 30;9(9):889. doi: 10.1038/s41419-018-0866-5 (PMC6117300; doi:10.1038/s41419-018-0866-5)
Supplement: Supplementary file 2 — Legends to Supplementary figures [file 41419_2018_866_MOESM2_ESM.doc]

**Supplementary Figure Legends**

**Figure S1** Trehalose induces autophagy and actives autophagic flux. (**a**) PC3 cells transiently transfected with GFP-LC3 were treated for 6, 12, 24 or 48 h with 100 mM trehalose. GFP WB analysis shows that the treatment with trehalose 100 mM for 48h decreases GFP-LC3-II expression, but the free GFP fragment is not detectable due to trehalose capability to induce its lysosomal degradation. In fact, the co-treatment of CQ (10 μM) and trehalose induces GFP-LC3 accumulation. (**b**) Filter retardation assay shows that retained polyQ insoluble species were decreased after trehalose treatment (100 mM) confirming that trehalose mediated autophagy activation is sustained by autophagic flux activation and protein degradation.

**Figure S2** Trehalose and rapamycin differently modify LC3/mitochondria co-localization in docetaxel-treated PC3 cells. PC3 cells were treated for 48 h with 100 mM trehalose or 100 nM rapamycin and/or 20 nM docetaxel. The cells were stained with 250 nM MitoTracker Orange for 30 min to stain the mitochondria and then fixed with paraformaldehyde. IF shows LC3 and mitochondria localization. Nuclei were stained with DAPI. Labelled cells were examined by confocal microscopy, scale bar 20 μm. These images confirmed and reinforced the results obtained by IF presented in Figure 5a.

**Figure S3** Trehalose and rapamycin differentially modify p62/mitochondria co-localization in docetaxel-treated PC3 cells. PC3 cells were treated for 48 h with 100 mM trehalose or 100 nM rapamycin and/or 20 nM docetaxel. The cells were stained with 250 nM MitoTracker Orange for 30 min to stain the mitochondria and then fixed with paraformaldehyde. IF shows p62 and mitochondria localization. Nuclei were stained with DAPI. Labelled cells were examined by confocal microscopy, scale bar 20 μm.

These images confirmed and reinforced the results obtained by IF presented in Figure 5b.

**Figure S4** Trehalose counteracts docetaxel-induced apoptosis in PC3 cells. Cells were treated with trehalose 100 mM and docetaxel 100 nM for 24 h. After treatment, cells were stained with Annexin V and PI. Dot-plots represent flow cytometry analysis of 10.000 events. Experiment was repeated three time and representative plots are shown. Flow cytometry was conducted using a NovoCyte 3000**.**

**Figure S5** Role of trehalose on docetaxel-induced cell death in DU145 cells. (a) DU145 cells were transfected with pCI-neo-hApg5-HA for 48 h or 72 h. ATG5 expression levels were analysed by WB analysis. (b) MTT viability assay performed on DU145 cells transfected with pCI-neo-hApg5-HA or pcDNA3 and treated with trehalose 100 mM and/or docetaxel 20 nM for 36 h. Six independent biological samples for each condition were analysed (n=6), bar graph represents the mean relative cell viability ± SD. Statistical analysis was performed using one-way ANOVA followed by Bonferroni post- test (* = p < 0.05).

**Figure S6** Schematic model of autophagy role in the docetaxel-response in CRPC.
